# Supplementary material for: Nondestructive flash cathode recycling
Source: Nat Commun. 2024 Jul 24;15:6250. doi: 10.1038/s41467-024-50324-x (PMC11269590; doi:10.1038/s41467-024-50324-x)
Supplement: Supplementary file 1 — Supplementary Information [file 41467_2024_50324_MOESM1_ESM.pdf]

Supplementary Information for

Nondestructive flash cathode recycling

Weiyin Chen,[1#] Yi Cheng,[1#] Jinhang Chen,[1#] Ksenia V. Bets,[2] Rodrigo V. Salvatierra,[1] Chang Ge,[3] John Tianci Li,[1] Duy Xuan Luong,[1,3] Carter Kittrell,[1,4,5] Zicheng Wang[1], Emily A. McHugh,[1] Guanhui Gao,[2] Bing Deng,[1] Yimo Han,[2] Boris I. Yakobson,[1,2,4*] and James M. Tour[1,2,3,4,5*]

[1]Department of Chemistry, Rice University, 6100 Main Street, Houston, Texas 77005, USA

[2]Department of Materials Science and NanoEngineering, Rice University, 6100 Main Street, Houston, Texas 77005, USA

[3]Applied Physics Program, Rice University, 6100 Main Street, Houston, Texas 77005, USA

[4]Smalley-Curl Institute, Rice University, 6100 Main Street, Houston, Texas 77005, USA

[5]NanoCarbon Center and the Rice Advanced Materials Institute, Rice University, 6100 Main Street, Houston, Texas 77005, USA

[#]These authors contributed equally.

*Corresponding author email: B.I.Y (biy@rice.edu); J.M.T (tour@rice.edu)

Supplementary Text

Abbreviations

CB	Carbon black
CEI	Cathode electrolyte interphase
CW	Cathode waste
FJH	Flash Joule heating
GHG	Greenhouse gas
Hydro	Hydrometallurgical
LCA	Life cycle analysis
LCO	Lithium cobalt oxide ($LiCoO_2$)
LIB	Li-ion battery
NMC	Lithium nickel-manganese-cobalt oxide ($LiNi_xMn_yCo_zO_2$, normally referred as NMC*xyz*, such as NMC811)
Pyro	Pyrometallurgical

Supplementary Note 1. Life cycle analysis.

1. Goal and scope.

The goal of this closed-loop life cycle analysis[61] is to consider and compare the potential economic and environmental effects in collection and treatment of the cathode wastes from the spent commercial lithium-ion batteries, and the resynthesis of new cathode materials. In particular, the GHG emission, energy consumption and water consumption are considered for the current recycling strategies, including hydrometallurgical method, pyrometallurgical method and direct recycling method, together with the flash recycling method as discussed in the work. The process to produce the cathode materials from virgin sources was also analyzed for comparison. The material transportation and GHG disposal cost are outside the scope of this limited study. The cost includes raw materials cost, manufacturing cost and fixed annual capital investment. The average energy cost is \$0.04 per kWh and the cost of water is \$0.005 per gallon. The wastewater discharge fee is \$0.007 per gallon. The fixed annual capital investment includes the operating labor, direct supervisory, laboratory charge, maintenance, and repairs. The manufacturing cost contains water and energy cost, the cost of GHG emission and wastewater discharge (~30% of fixed annual capital investment) and general expense (~20% of manufacturing cost). Overhead costs (~20% of product cost) are also considered.

2. Scenario description, system boundaries, and inventory analysis.

Five scenarios, hydrometallurgical method, pyrometallurgical method, direct recycling method, virgin cathode production, and flash recycling method were considered in this part (**Fig. 5**). In all the scenarios, treating 1 kg of spent lithium-ion batteries was used to evaluate and normalize the material and energy flow according to the life cycle inventory, which is summarized

in Supplementary Table 5. A cradle-to-gate LCA does not consider the use of the cathode materials nor their disposal (grave) since it was assumed that new cathode materials and recycled cathode materials had the same, if not better, usage and recycling stages.

Scenario 1. Hydrometallurgical method: The process discussed here was modified based on the previous work[17]. In this scenario, the spent lithium-ion batteries (1 kg) were crushed and shredded after discharge pretreatment for safety considerations. Then, the battery pieces were calcined at 873 K for 2 h to decompose the binders, electrolytes, and the formed impurities during electrochemical cycling, which was mainly the organic SEI components atop the cathode particles The compact SEI layer could hinder the kinetics of the acid leaching process. The energy consumption for low temperature calcination was estimated based on the pilot-scale recycling process using commercial furnace reported in previous work[1], whose temperature, power, and mass loading were ~873 K, 6 kW and 227 kg, respectively. The same below. The wet granulation, density separation and froth flotation were used to remove other battery components and to collect the spent cathode powder. The separation treatment can reduce the water consumption and total acid amounts in the acid leaching step. Note that the acid amount used in the leaching step was calculated based on the mass of the solid and the pulp density (~2%). The concentration of the acid was set as 4 mol L^{-1} (HCl solution) due to the low efficiencies with dilute acids. The metal impurities in the leachate, including Cu and Al are removed through a series of pH adjustments. Then the ratio of Ni, Mn, and Co is tailored to the desired ratio by adding virgin metal sulfates as needed. Subsequently, the adjusted metal sulfate solution undergoes the co-precipitation reaction under nitrogen, and the pH value is controlled between 10-11 to produce transition metal hydroxide precursor powder. Then, 1 mol of the precursor is mixed with 1.05 mol of lithium carbonate.

Finally, the mixture is sintered at 723 K for 5 h and 1173 K for 14 h. The temperature, power, and mass loading were ~1173 K, 45 kW and 245 kg, respectively. After calcination, recovered cathode power is ready for use in new batteries, enabling a closed-loop approach for the cathode materials. The GHG emission and energy consumption for individual steps were estimated from the Everbatt 2020 software and Argonne GREET model, the same below.

Scenario 2. Pyrometallurgical method: In this scenario, the spent batteries were directly smelted after discharge pretreatment. The temperature was ~1873 K and the duration is ~3 h. The purpose of high temperature smelting is to decompose all the organic impurities and thermally reduce the transition metal oxides to their metal counterparts. The Li salts would result in the formation of the slag with Al and Ca as the byproduct. Recent work has shown the Li species can be recycled by post-treating/activating the slag or evaporating the Li species during the temperature smelting process[45]. The energy consumption for high temperature smelting was estimated based on the pilot-scale recycling process using the commercial furnace, whose temperature, power, and mass loading were ~1873 K, 36 kW and 174 kg, respectively. The exhaust gas could be toxic to the crew and harmful to the environment, therefore a gas treatment was applied followed by the high temperature smelting process. Note that the acid amount used in the leaching step was calculated based on the mass of the solid and the pulp density (~5%). The Li species could be collected from the slag after the activation process as demonstrated in the recent work, and the post-treatment of the by-products, such as slag here is not considered in the analysis. The reduced transition metal chunks could dissolve in dilute acids with a high leaching efficiency as discussed in the previous work, therefore the concentration of the acid to dissolve the matte was set as 1 mol L^{-1} (HCl solution). It was assumed that subsequent operations related to treatment of

the leachate and the post-synthesis of the cathode materials were similar to the hydrometallurgical method as mentioned in Scenario 1.

Scenario 3. Direct recycling method: In this scenario, the spent batteries were shredded to collect the spent cathode waste after discharge pretreatment. Some work also used 1-methyl-2-pyrrolidinone to soak the cathode side for ~6 h to remove the poly(vinylidene fluoride) binder[21]. Here, a one-step low temperature calcination at 873 K for 2 h was applied to remove the polymer binder, electrolytes, and the formed impurities during electrochemical cycling, which was mainly the organic SEI components atop the cathode particles. The energy consumption for low temperature calcination was estimated based on the pilot-scale recycling process using a commercial furnace, whose temperature, power, and mass loading were ~873 K, 6 kW and 227 kg, respectively. After the pretreatment, the mixture was screened to collect the metal case and plastics. The anode powder was separated by froth flotation. And the cathode powder was mixed with Li_2CO_3 to achieve the molar ratio n(Li)/n(TM) = 1.10. Here TM includes cobalt, nickel, and manganese. If the cathode waste includes battery metals, such as aluminum or iron, the corresponding stoichiometric ratio should also be considered. The mixture was heated at ~1073 K for 12 h to relithiate the cathode powder and restore the structure. The energy consumption for high temperature calcination was estimated based on the commercial furnace, whose temperature, power, and mass loading were ~1073 K, 40 kW and 245 kg, respectively. The relithiated cathode powder was regarded as the final product from direct recycling method.

Scenario 4. Virgin cathode production: In this scenario, the same amount (~0.35 kg) of the new cathode materials from mining the virgin ores are considered as a comparison. Based on the Everbatt 2020 software[17], the water consumption, energy consumption and the GHG emissions for

the cathode production were 56.22 L, 91.00 MJ and 6.24 kg, respectively. And the estimated cost was ~$13.39.

Scenario 5. Flash recycling method: In this scenario, the spent batteries were disassembled and scrapped to collect the spent active materials. The disassembly step can be achieved by a commercial core drill with a silicon carbide blade, which can reduce the manual disassembly cost. The spent cathode materials can be directly used as the reactants for the flash recycling process without any pretreatment. The energy consumption was estimated based on the total energy output from the capacitors. Here the specific energy density was 0.31 kWh kg^{-1}. Followed by magnetic separation, the magnetic portion of the flash-recycled cathode material was mixed with Li_2CO_3 to achieve the molar ratio n(Li)/n(TM)=1.10. It was assumed that subsequent operations related to the post-synthesis of the cathode materials were like the direct recycling method as mentioned in Scenario 3. The weight of the final cathode was ~0.35 kg. The prospective cradle-to-gate LCA was used for each scenario to consider the economic and environmental impacts from the recycling of the spent lithium-ion batteries to all reaction processes involving the production of ~0.35 kg cathode materials from spent lithium-ion batteries.

For environmental impacts, we considered the energy and waste consumption, and greenhouse gas emission for different recycling methods. We assume that 1 MJ electricity produces 0.13 kg GHG and 0.67 L water, which comes from GREET 2020 (ref. 17). The energy consumption will come from electricity usage during the cathode relithiation, FJH process, and spent battery disassembly.

Cathode relithiation requires sintering the cathode power and extra lithium salt at 1073 K for 12 h, which is the same as the direct recycling method. A commercial furnace can be used here, and the parameters include temperature, power, and mass loading of ~1073 K, 40 kW and 245 kg,

respectively. Therefore, the energy consumption is 7.05 MJ per kg cathode power. The water consumption for cathode relithiation is based on the above relationship, which is (7.05×0.67) L = 4.726 L. Besides the GHG produced from using electricity, the decomposition of Li_2CO_3 salt can also produce CO_2, which accounts for 0.118 kg GHG emission. Therefore, the total GHG emission for cathode relithiation is (7.05×0.13+0.118) kg = 1.035 kg.

Similarly, the electricity usage for the FJH process is 0.31 kWh kg^{-1}; here we consider 10% overhead as well for reaction atmosphere and the total energy is (0.31×3.6×1.1) MJ = 1.23 MJ. The water usage and GHG emission for the FJH process is calculated based on the above relationship. Therefore, the water usage is 0.825 L and GHG emission is 0.160 kg, respectively.

The disassembly step can be achieved by a commercial core drill with a silicon carbide blade, and scrapping is calculated based on Everbatt 2020 (ref. 18). Since there is no extra water required and no extra GHG emission, the energy consumption is used to estimate the water usage and GHG emission in this step. The electricity consumption is estimated to be (29.828+149.14) kWh for 1740 kg spent batteries, corresponding to 0.38 MJ per kg spent batteries. Therefore, the water usage is 0.26 L and GHG emission is 0.050 kg, respectively.

The same discharge step is used to estimate the energy and waste consumption, and greenhouse gas emission as shown in other recycling methods. The average water assumption is 0.5 L per kg spent batteries. The energy consumption is estimated based on the usage of conveyor, which is ~0.03 MJ per kg spent batteries. Therefore, the water usage is (0.50+0.03×0.67) L = 0.52 L and GHG emission is (0.03×0.13) kg = 0.004 kg, respectively.

3. Life cycle impact assessment.

In this study, the environmental impacts were categorized into 5 midpoint indicators, including water consumption (Fig. 5e), GHG emissions (Fig. 5g), and energy consumptions (Fig.

5f), concentrated 12 M HCl consumption (Fig. 5d) and the estimated cost (Fig. 5h) in treating 1 kg of spent lithium-ion batteries.

4. Sensitivity and uncertainty.

Due to the data availability, the energy consumption, GHG emission and water consumption values for various processes involved in this study were from different sources, which could introduce some uncertainty. Secondly, several assumptions were made in this study with regard to the low temperature calcination or high temperature smelting process, the production of recycled cathode materials. Thirdly, even though we proposed the scalability of the FJH activation process and calculate the energy based on 0.44 kWh kg^{-1}, the FJH process realized in this work was on the gram scale; hence, there might be uncertainty in energy consumption when scaling up the FJH activation method to ton scale.

Supplementary Note 2. Structure value estimation

The purpose to estimate the structure value of the cathode materials is to highlight the importance of nondestructive recycling strategies. The three-dimensional morphology of the cathode materials can be estimated based on the price difference between the cathode materials and the sum of the individual elements as shown in the following equation,

$$M(structure\ value) = M(ABC) - \sum_{i=A}^{C} \alpha M(i) \quad \text{(S1)}$$

M(structure value), M(ABC), M(i) mean the unit prices of structure, compound ABC, and their elements, respectively. α is the mass ratio of each element in the compound.

For example, the structure value of $LiCoO_2$ is,

$$M(structure\ value) = M(LiCoO_2) - \frac{7}{98}M(Li) - \frac{59}{98}M(Co) - \frac{32}{98}M(O_2) \quad \text{(S2)}$$

If *M(structure value)* > 0, the three-dimensional morphology has a positive value to the cathode materials. Otherwise, the three-dimensional morphology has a negative value to the cathode materials.

Supplementary Note 3. SAFETY NOTES[31]

FJH involves high currents and voltages, which has a risk of electrical shock or even electrocution, so these features should be implemented. This list is not intended to be comprehensive, but demonstrative of the protocols needed to minimize risk.

1. Enclose or carefully insulate all wire connections.
2. All connections, wires and components must be suitable for the high voltages and currents.
3. Be aware that component failure could cause high voltage to appear in unexpected places, such as heat sinks on the switching transistors.
4. Control wires should have opto-isolators rated for high voltage.
5. Provide a visible charge indicator. A 230 V clear glass incandescent light bulb is a good choice as the glow on the filament also provides an approximate indicator of the amount of charge on the capacitor bank. Bright light = danger!
6. Do not use toggle switches with metal toggles. If an arc develops, the metal toggle could become charged.
7. One hand rule. Use only one hand when working on the system, with the other hand not touching any grounded surface.
8. Install bleed resistors in the range of 100,000 ohms on each capacitor so that charge will always bleed off in ~1 h.

9. Provide a mechanical discharge circuit breaker switch connected to a power resistor of a few hundred ohms to rapidly bleed off the capacitor charge.
10. Provide a "kill" circuit breaker switch to disconnect the sample holder from the capacitor bank.
11. Provide an AC disconnect circuit breaker switch.
12. Post a high voltage warning signs on the apparatus.
13. Use of circuit breakers as switches. Circuit breakers have built-in arc suppression that can interrupt 1000 amps or more. Conventional switches do not have such a high level of arc suppression and can burn out or weld closed due to the high current pulses.
14. Use circuit breakers rated for DC voltage. Most AC circuit breakers have a DC rating 1/2 the voltage or less since DC arcs are much more difficult to suppress. Circuit breakers designed for DC solar power systems are a good choice.
15. When choosing circuit breakers, choose by the time curves typical for 0.1 s, rather than the steady state current rating. K-type DC circuit breakers will have ~10× higher trip current at 0.1 s compared to their rated current, and Z-type breakers will have ~4× higher trip current at 0.1 s. This "delayed trip" designed into most circuit breakers will allow much higher pulse currents than the steady state rating of the breaker.
16. Include a small amount of inductance in the discharge circuit to limit the rise time to a millisecond or more. Extremely fast discharges can damage components and cause RF interference with other lab apparatus.
17. Keep in mind that the system can discharge many thousands of Joules in milliseconds, which can cause components such as relays or even capacitors to explode. These

components should be enclosed to protect against both high voltage and possible flying debris.

18. Keep a voltmeter with high voltage test leads handy at all times. When working on the capacitor bank, always check the voltage on each. A broken wire or loose connection could leave the capacitor in a charged state.
19. Wear electrically approved thick rubber gloves extending to the elbows when using the apparatus to protect from electrocution.
20. All users should be properly trained by an experienced electrical technician.
21. Have a qualified electrical engineer inspect the instrument for safety before its use and have it reinspected weekly.

Supplementary Note 4. Discussion of re-flashing the nonmagnetic portion

The remaining ~10 wt% of flash product, derived from spent CW, which is not captured by the magnet can be combined with minor portions from other FJH runs to be re-flashed, and the flash Joule heating condition is the same as the one used for cathode waste in the Experimental Section. Here, the small batch experiments are used as the demonstration. Thereby, ~60 wt% of this can be magnetically recovered. And it is similar in behavior to the original FJH-CW as shown in Supplementary Figs. 5 and 6. The ICP-OES results show good recovery yields from the re-flash process, including Li (79%), Co (77%), Ni (73%) and Mn (84%). Therefore, further use of the remaining 10 wt% of the nonmagnetic portion in the re-flash recycling process can achieve a high recovery yield for all the valuable metals, including Li (92%), Co (93%), Ni (96%) and Mn (98%) for spent CW.

Supplementary Note 5. Recovery efficiency calculation

Recovery efficiencies of the different battery metals in the FJH-CW are very important for flash recycling method, which can be evaluated by the metal recovery yield (α),

$$\alpha = \frac{m(N,FJH-CW)}{m(N,CW)} \times 100\% \quad \text{(S3)}$$

The $m(N, FJH - CW)$ and $\mathrm{m(N, CW)}$ represent the amount of studied species N in FJH-CW and CW reactant, respectively.

The amounts are determined by the ICP-OES or ICP-MS and calculated below,

$$\frac{m(N,FJH-CW)}{m(N,CW)} = \frac{C(N,FJH-CW)}{C(N,CW)} \times \frac{m_1(N,FJH-CW)}{m_1(N,CW)} \times \frac{m_2(N,CW)}{m_2(N,FJH-CW)} \times \beta \quad \text{(S4)}$$

The $C(N, FJH - CW)$ and $C(N, CW)$ represent the mass concentration of species N in the diluted solutions derived from FJH-CW and CW reactant, respectively. The $m_1(N, FJH - CW)$ and $m_1(N, CW)$ represent the mass of the diluted solutions derived from FJH-CW and CW reactant. The $m_2(N, CW)$ and $m_2(N, FJH - CW)$ represent the mass of sample used in the ICP-OES or ICP-MS experiment. β is the flash Joule heating reaction yield.

$$\beta = \frac{m_3(N,FJH-CW)}{m_3(N,CW)} \quad \text{(S5)}$$

The $m_3(N, CW)$ and $m_3(N, FJH - CW)$ represent the total mass of CW reactant and FJH-CW, respectively.

Flash Joule Heating Setup

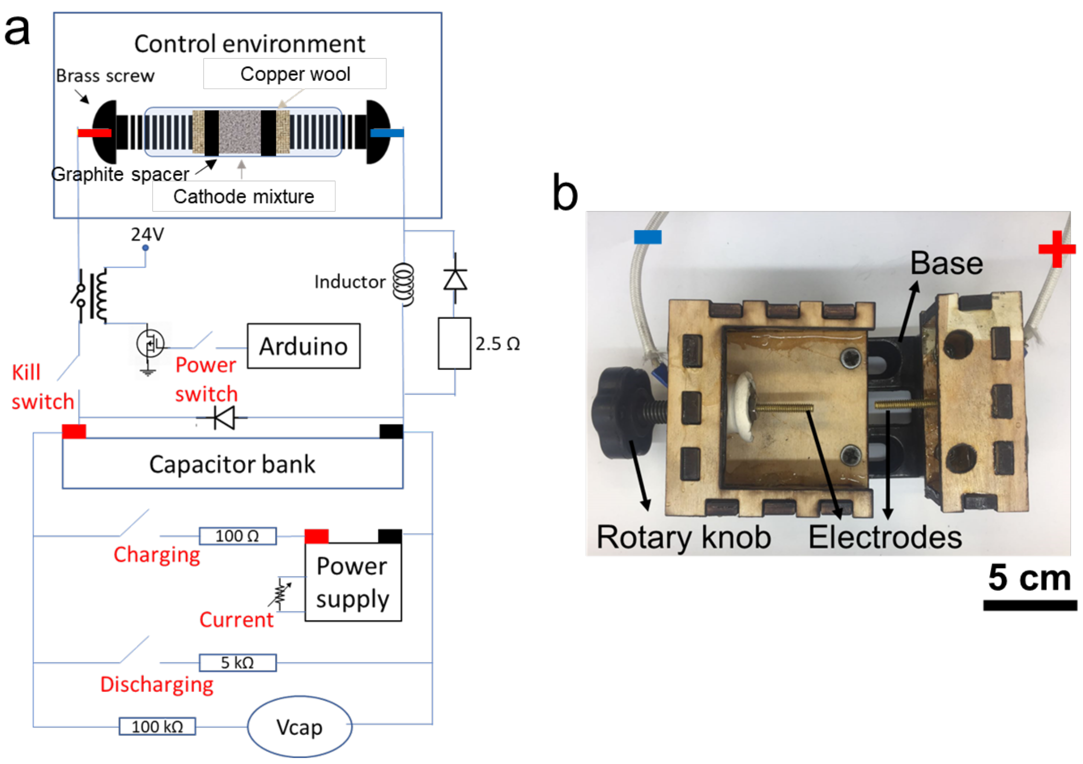

Supplementary Fig. 1. The scheme of FJH system. a, Electrical schematic of the FJH system. **b,** The photograph of FJH reaction box.

10 aluminum electrolytic capacitors (450 V, 6 mF, Mouser #80-PEH200YX460BQU2) with a total capacitance of 60 mF are used for charging in the small batch (~200 mg per batch). Additional 4 aluminum electrolytic capacitors (450 V, 16 mF) with a total capacitance of 132 mF are used for charging in the larger batch (~800 mg per batch). The brass screw electrodes do not fit snugly into the quartz tube. This permits the slight outgassing of the excess carbon compounds during the flash Joule heating process. An Arduino controller relay with programmable millisecond-level delay time is used to control the discharge time, and the electric energy is provided by the capacitor bank. Safety glasses designed for welding are generally suitable and

recommended during the flash reaction because they effectively block infrared as well as ultraviolet light. More safety guidelines can be found in Supplementary Note 3 (ref. *9*).

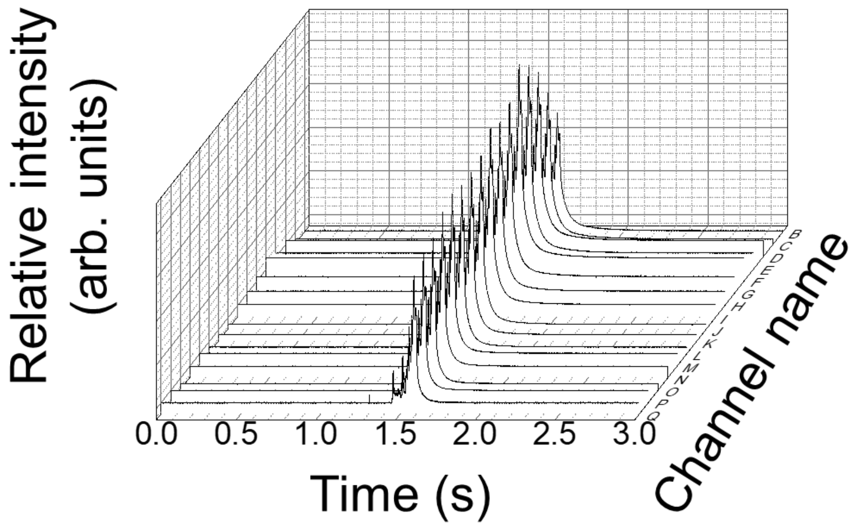

Supplementary Fig. 2. The spectra recorded by the home-built spectrometer with 16-channel optical fibers. The reaction temperature during the flash Joule heating process. The wavelengths of these channels range from 1000 nm to 640 nm with equal intervals of 24 nm. Black body radiation (BBR) fitting is subsequently used to obtain the temperature at each time point as shown in **Fig. 1e**.

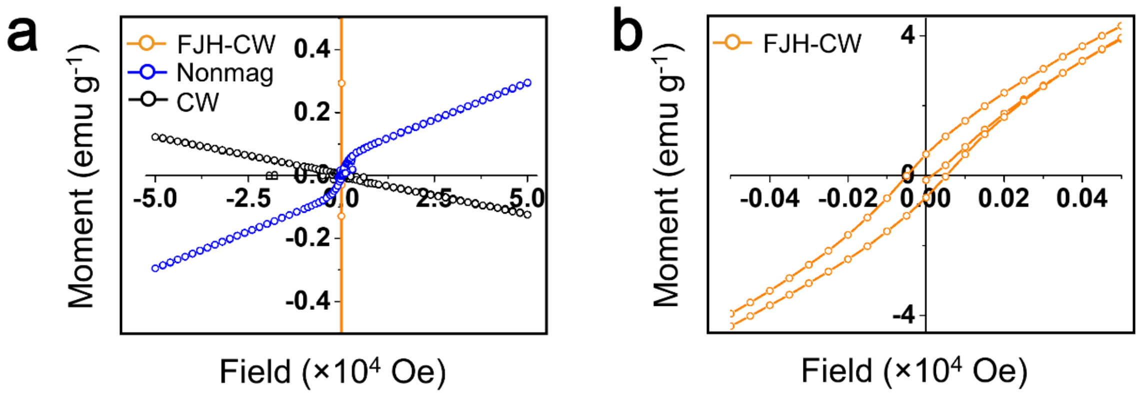

Supplementary Fig. 3. Magnetic response of flash products derived from spent CW. a, The magnified hysteresis loop for cathode waste (CW, black), ferromagnetic portion of flash Joule heating cathode waste (FJH-CW, orange) and the non-ferromagnetic portion (nonmag, blue). **b,** The behavior of the hysteresis loop around the origin for the FJH-CW.

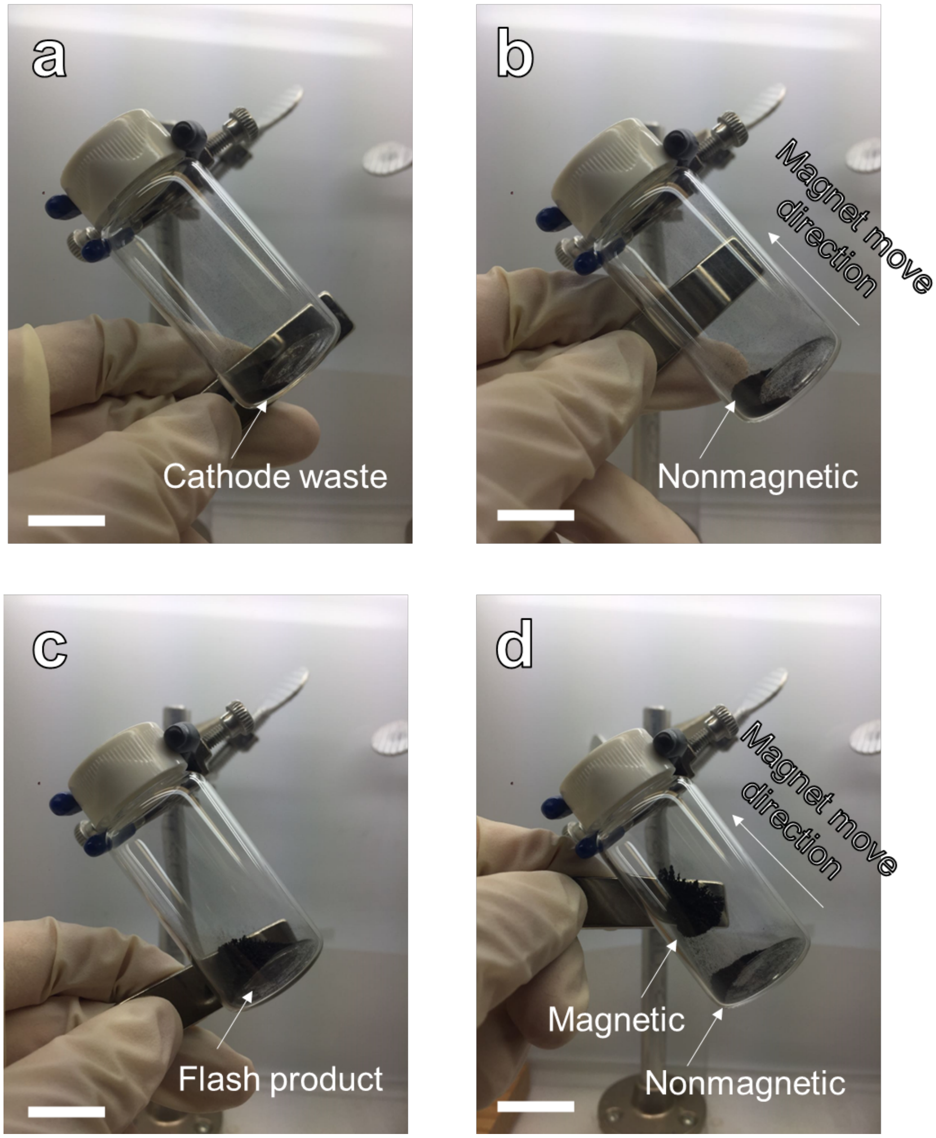

Supplementary Fig. 4. Magnetic response of cathode waste and flash product. a,b, The photos showing that the cathode waste is not attracted by a bar magnet, whereas **c,d,** show that the

ferromagnetic portion of flash Joule heating cathode waste can be separated by the bar magnet. The scale bars are 2 cm for all the photos.

As displayed in **Fig. 1g** and Supplementary Fig. 3, the ferromagnetic portion of the flash Joule heating cathode waste (FJH-CW, orange curve) has a sharp response to the external magnetic field (~10 emu g^{-1} at 1900 Oe) and the magnetic moment reaches saturation (~17 emu g^{-1}) at 8000 Oe. This magnetization is strong enough to ensure the effective separation of the ferromagnetic portion by a normal hand-held magnet with magnetic field strength ~5000 Oe. The coercivity force, as calculated from Supplementary Fig. 3b, is small, which indicates that the material's magnetization can easily reverse direction without dissipating significant energy (hysteresis losses). Contrastingly, the non-ferromagnetic portion (blue curve) and the intrinsic CW (black curve) show a weak magnetic response to the external magnetic field, and they are paramagnetic and diamagnetic materials, respectively. Therefore, a normal magnet can be used to capture the ferromagnetic portion of flash Joule heating cathode waste to reclaim the reaction precursors, as shown in Supplementary Fig. 4, for the subsequent cathode resynthesis.

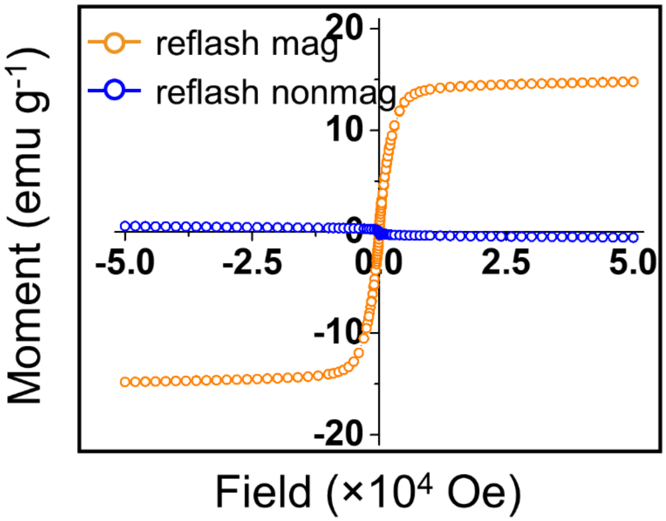

Supplementary Fig. 5. Magnetic response of re-flashed cathode materials. The magnetic response of re-flashed cathode waste ferromagnetic portion (orange) which is ~60 wt% of the product and re-flashed cathode waste non-ferromagnetic portion (blue) which is ~40 wt% of the product. The similar magnetization behavior of re-flashed cathode waste ferromagnetic portions ensures the effective separation of them by the same magnet with magnetic field strength ~5000 Oe.

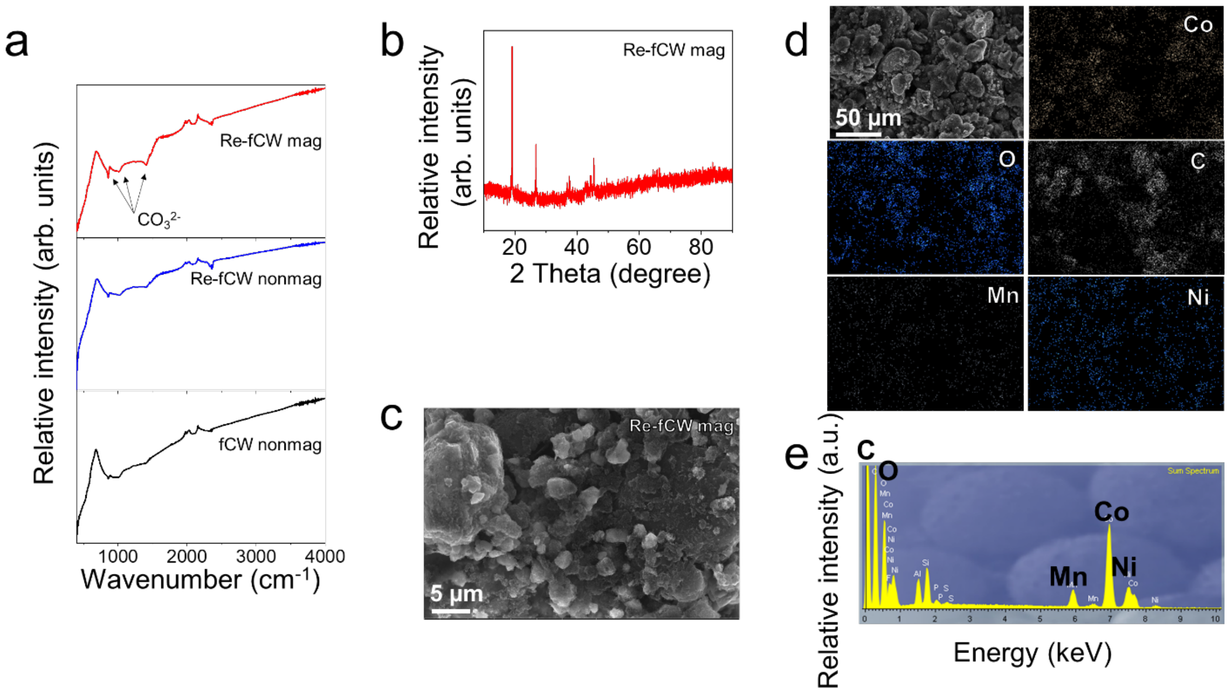

Supplementary Fig. 6. Structures and chemical components of re-flash recycled products. a, The FTIR spectra of nonferromagnetic portion of the flashed product derived from CW (black), ferromagnetic portion of the re-flashed CW (red) and nonferromagnetic portion of the re-flashed CW (blue). **b,** The XRD spectra of ferromagnetic portion of the re-flashed CW. **c,** SEM images of ferromagnetic portion of the re-flashed CW. **d,** The energy dispersive analysis element mapping and **e,** corresponding spectrum of the ferromagnetic portion of the re-flashed CW.

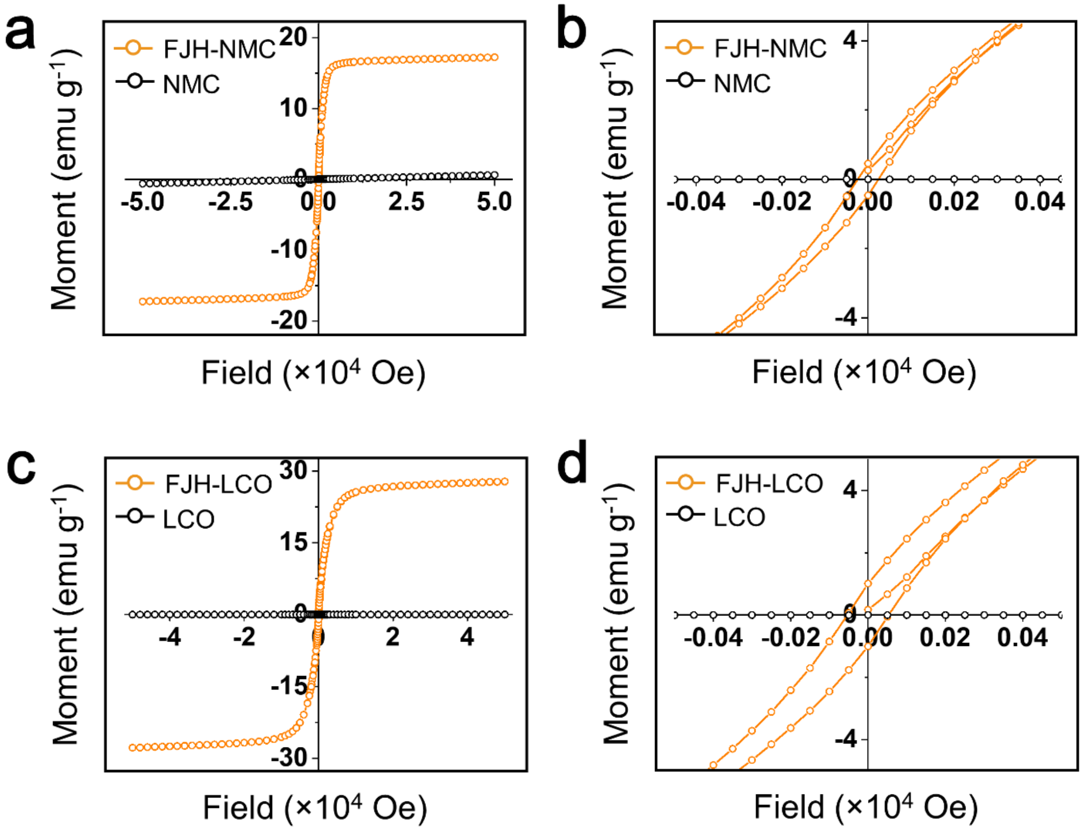

Supplementary Fig. 7. Magnetic response of various cathode materials. a, The room temperature (300 K) hysteresis loops for NMC (black curve) and the ferromagnetic portion of flashed NMC (FJH-NMC, orange curve). **b,** The behavior of the hysteresis loop around the origin for NMC and FJH-NMC. **c,** The room temperature (300 K) hysteresis loops for LCO (black curve) and the ferromagnetic portion of flashed LCO (FJH-LCO, orange curve). **d,** The behavior of the hysteresis loop around the origin for LCO and FJH-LCO.

Different categories of cathode materials are used to test the feasibility of the method, such as LCO and NMC, and the corresponding magnetic test results are reported in Supplementary Fig. 7. In both cases, the ferromagnetic portions of the flashed products show a sharp response to the external magnetic field while the reactants have no ferromagnetic response. This magnetization is

strong enough to ensure the effective separation of the ferromagnetic portion by a normal hand-held magnet with magnetic field strength ~5000 Oe.

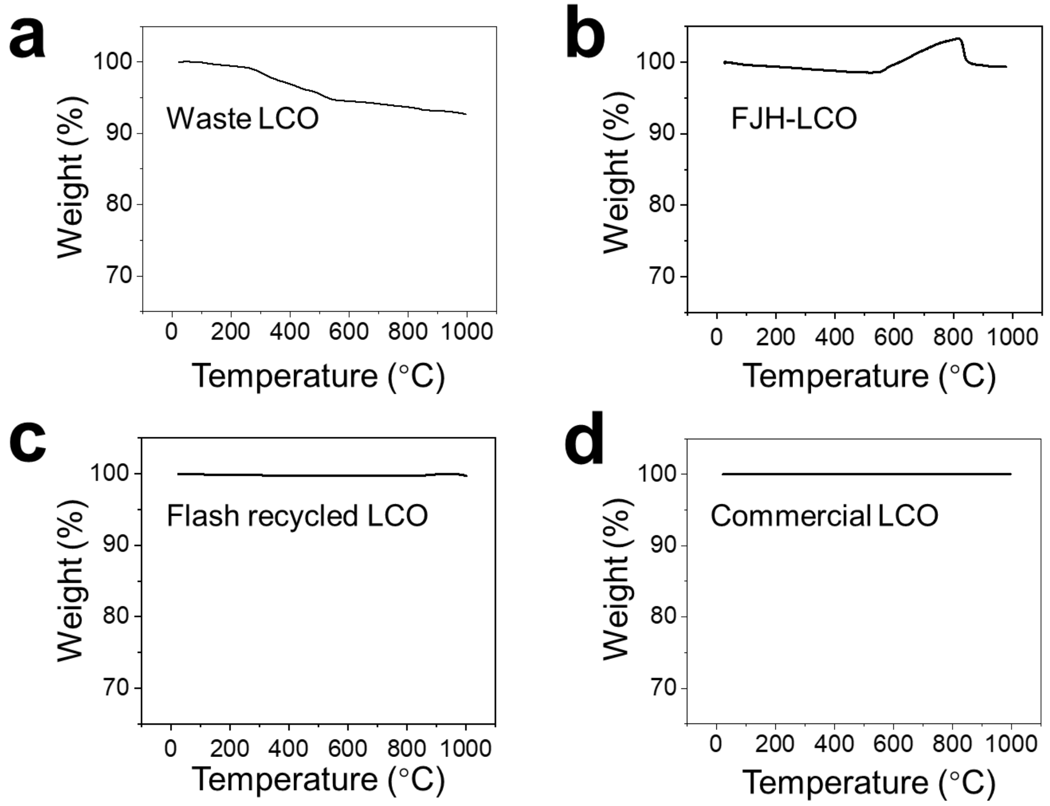

Supplementary Fig. 8. TGA results of various cathode materials. a, Waste LCO. **b,** FJH-LCO. **c,** Flash recycled LCO. The slight weight gain may be due to oxidation. **d,** Commercial LCO. TGA data were collected at a heating rate of 10 °C/min under air. The air flow was set to 80 mL/min.

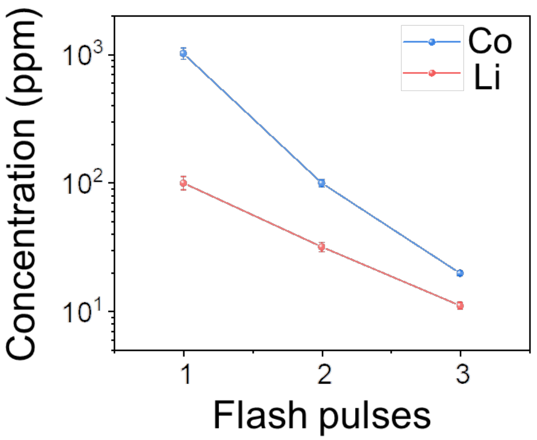

Supplementary Fig. 9. The relationship between the flash pulses and the concentration of battery metals within the nonmagnetic portion of the flashed products derived from waste LCO. The error bars reflect the standard deviations from at least three individual measurements.

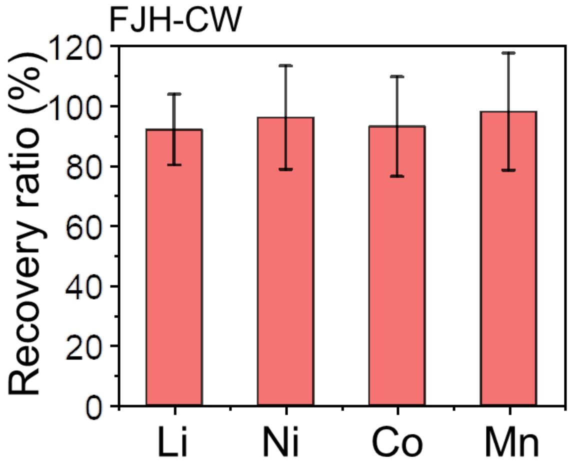

Supplementary Fig. 10. Recovery efficiencies of various battery metals. Recovery yields of Li, Co, Ni, and Mn in the ferromagnetic portion of flash Joule heating cathode waste (FJH-CW). The error bars reflect the standard deviations from at least three individual measurements.

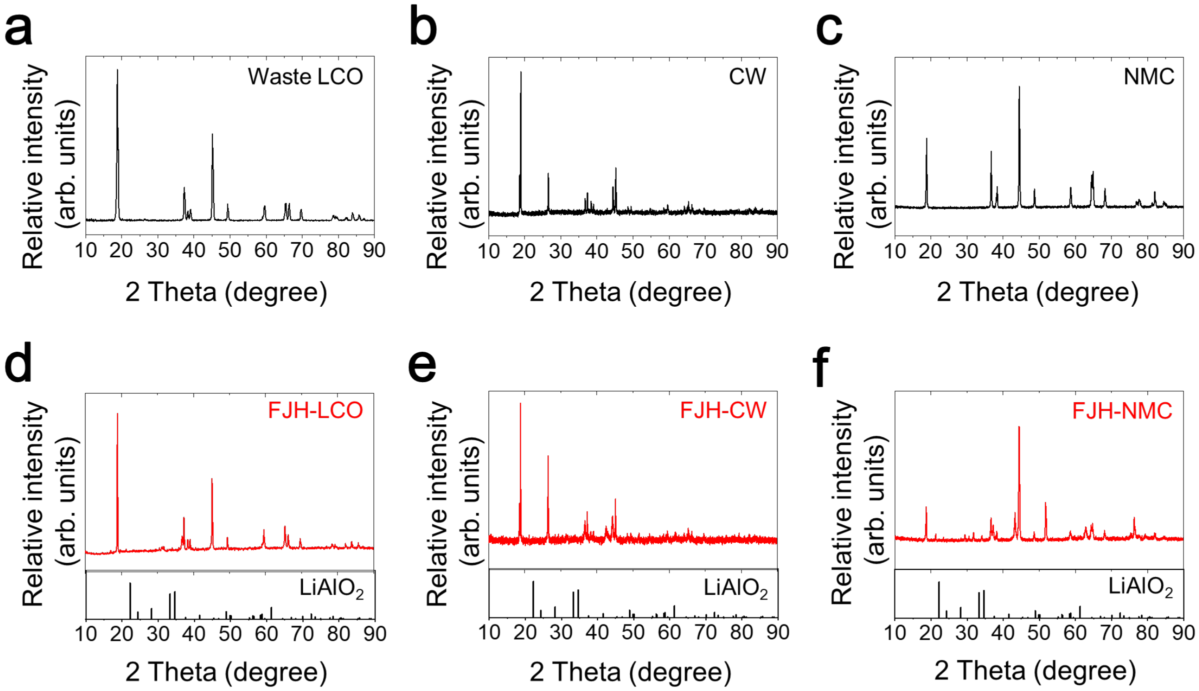

Supplementary Fig. 11. XRD results of various cathode materials before and after FJH treatment. a, Waste LCO. **b,** CW. **c,** NMC. **d,** FJH-LCO. **e,** FJH-CW. **f,** FJH-NMC. Powder diffraction file: 38-1464, $LiAlO_2$.

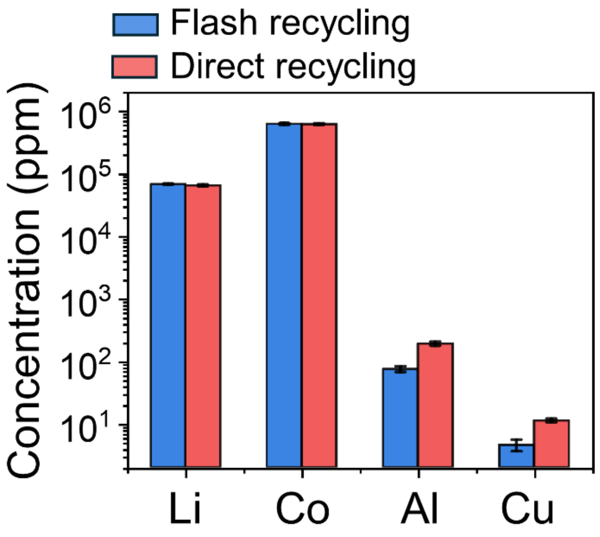

Supplementary Fig. 12. ICP-MS results of recycled cathode materials. The error bars reflect the standard deviations from at least three individual measurements.

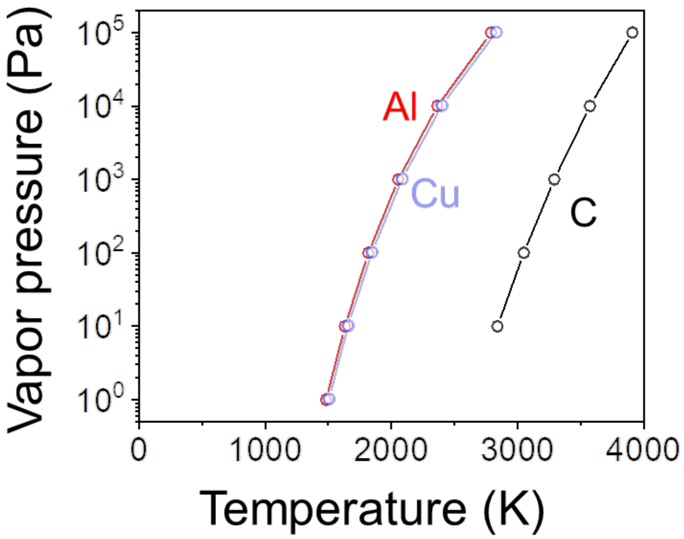

Supplementary Fig. 13. Vapor pressure–temperature relationship of metal impurities and carbon.

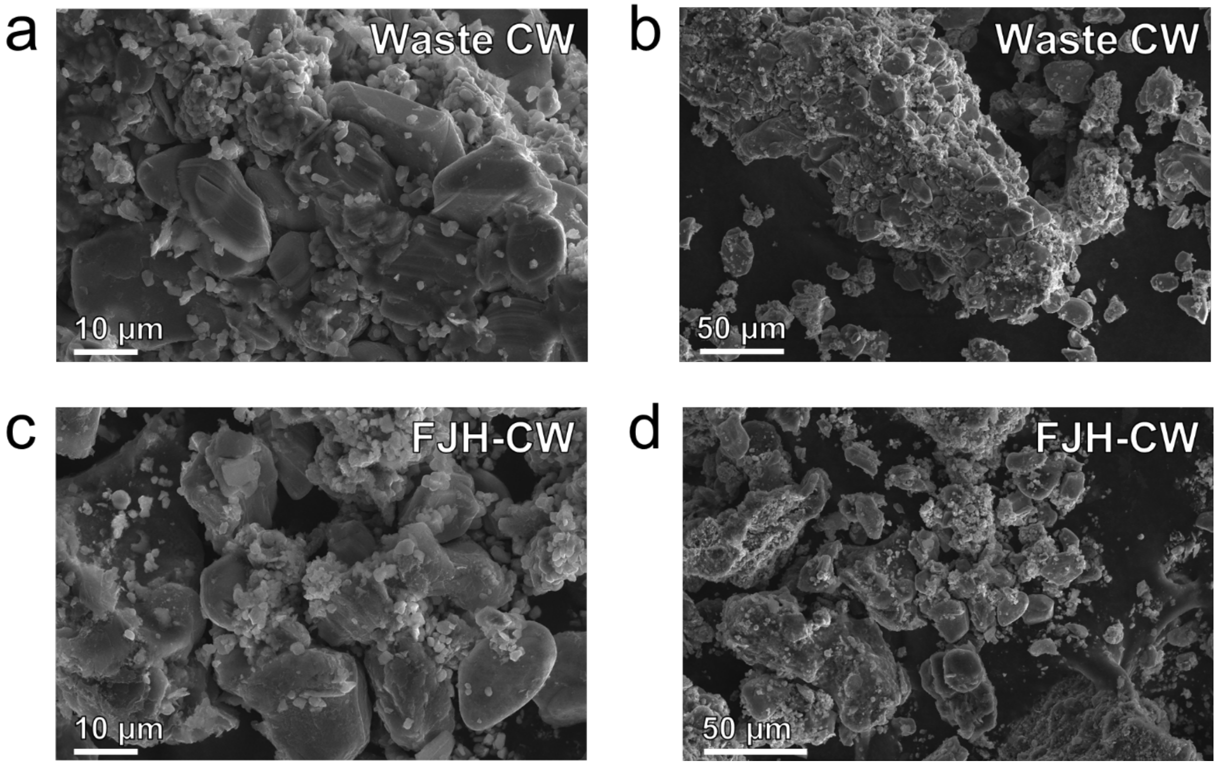

Supplementary Fig. 14. SEM images of cathode waste before and after FJH treatment. a,b, CW. **c,d,** FJH-CW.

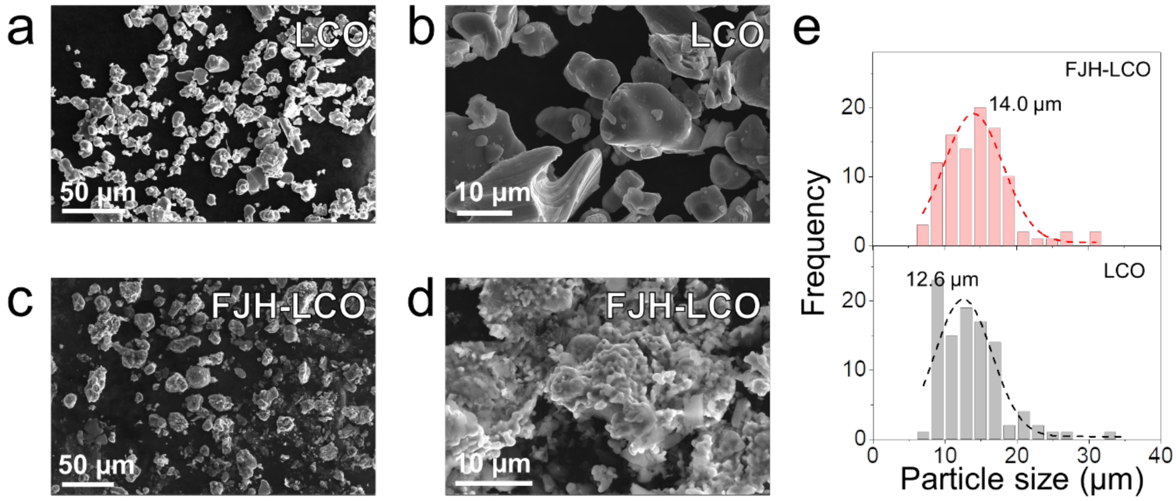

Supplementary Fig. 15. SEM images of new LCO before and after FJH treatment. a,b, LCO. **c,d,** FJH-LCO. **e,** Size distribution for the particles.

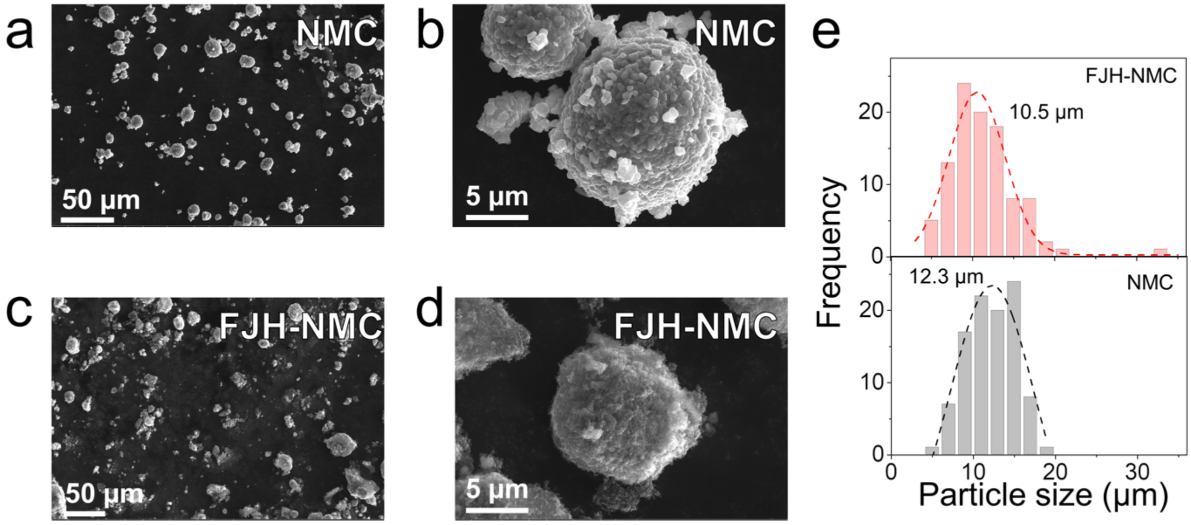

Supplementary Fig. 16. SEM images of new NMC before and after FJH treatment. a,b, NMC. **c,d,** FJH-NMC. **e,** Size distribution for the secondary particles.

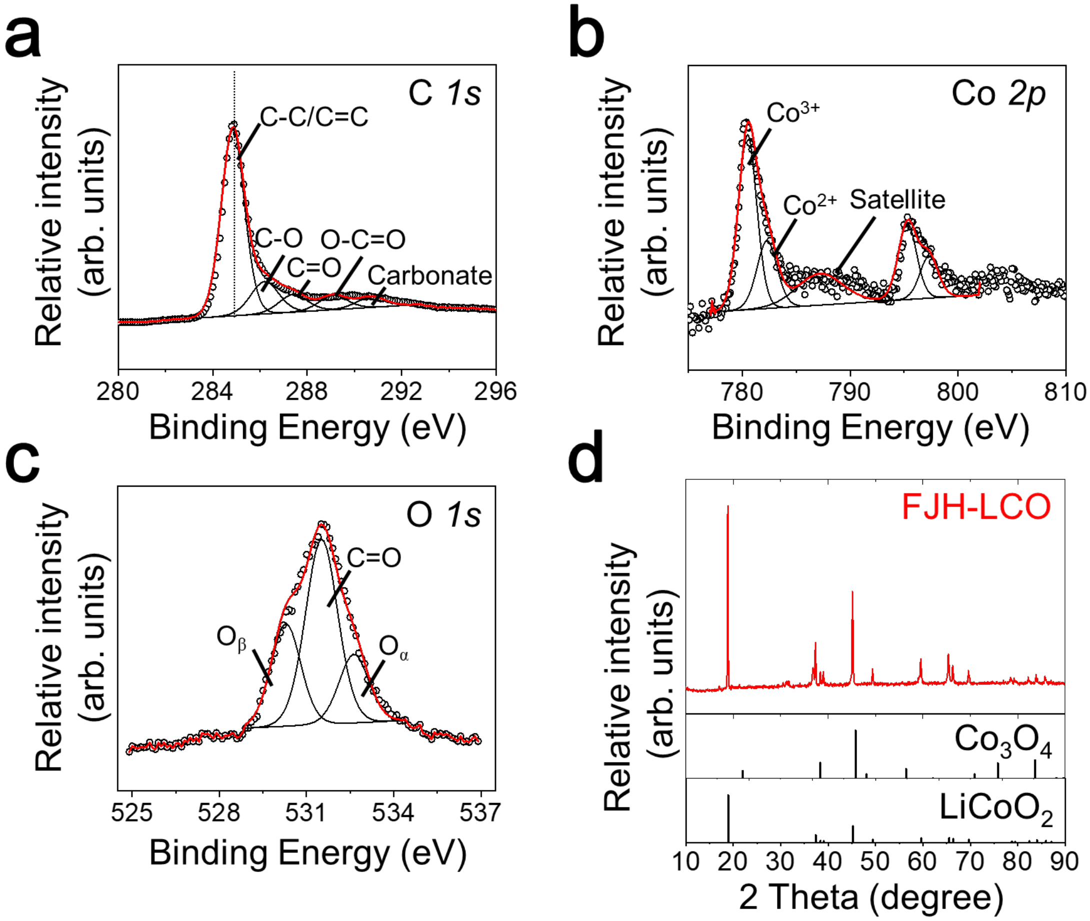

Supplementary Fig. 17. The high-resolution elemental analysis of new LCO after FJH treatment. a, C *1s*. **b,** Co *2p*. **c,** O *1s*. **d,** XRD result. Powder diffraction file: 43-1003, Co_3O_4. 16-0427, $LiCoO_2$.

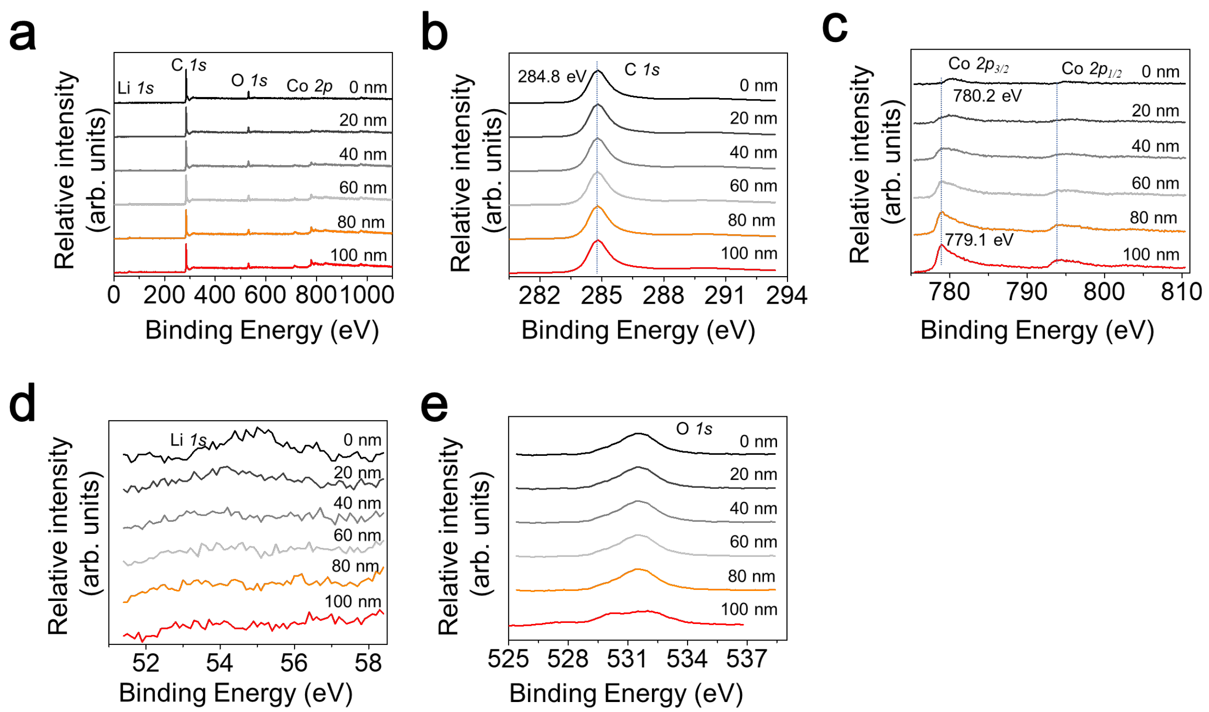

Supplementary Fig. 18. XPS analysis of new LCO after FJH treatment at different depths. **a,** The full scan XPS result of the ferromagnetic FJH-nLCO. High resolution XPS spectra of **b,** C *1s*, **c,** Co *2p*, **d,** Li *1s*, and **e,** O *1s* at the surface and subsurface area of FJH-nLCO. Spectra were acquired at different depths after surface etching (see Materials and Methods Section). The Co $2p_{3/2}$ peak shifts to higher binding energy, indicating the surface reduction after FJH treatment.

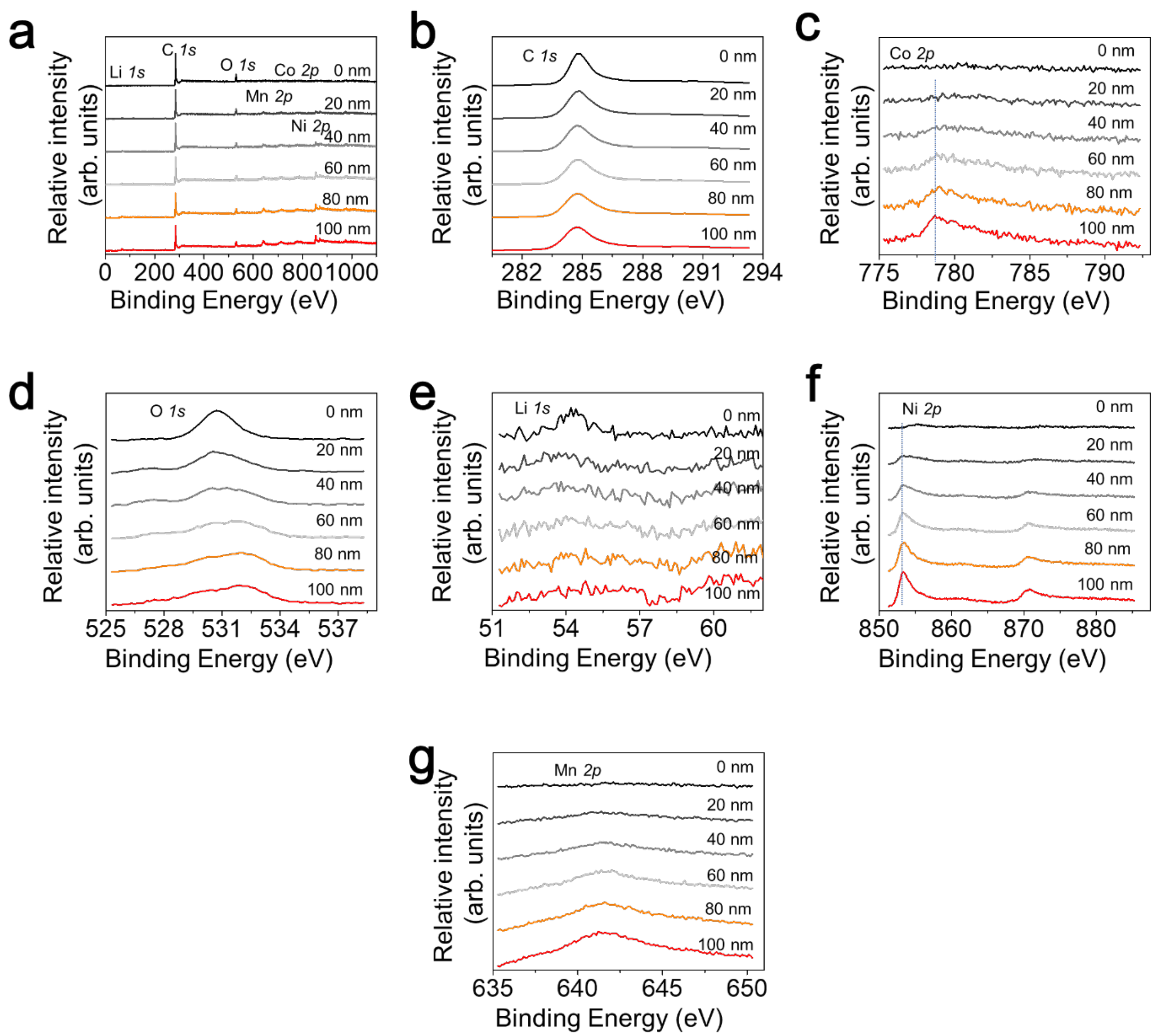

Supplementary Fig. 19. XPS analysis of new NMC after FJH treatment at different depths. **a,** The full scan XPS result of the ferromagnetic FJH-nNMC. High resolution XPS spectra of **b,** C *1s*, **c,** Co *2p*, **d,** O *1s*, **e,** Li *1s*, **f,** Ni *2p* and **g,** Mn *2p* at the surface and subsurface area of FJH-nNMC. Spectra were acquired at different depths after surface etching (see Materials and Methods Section).

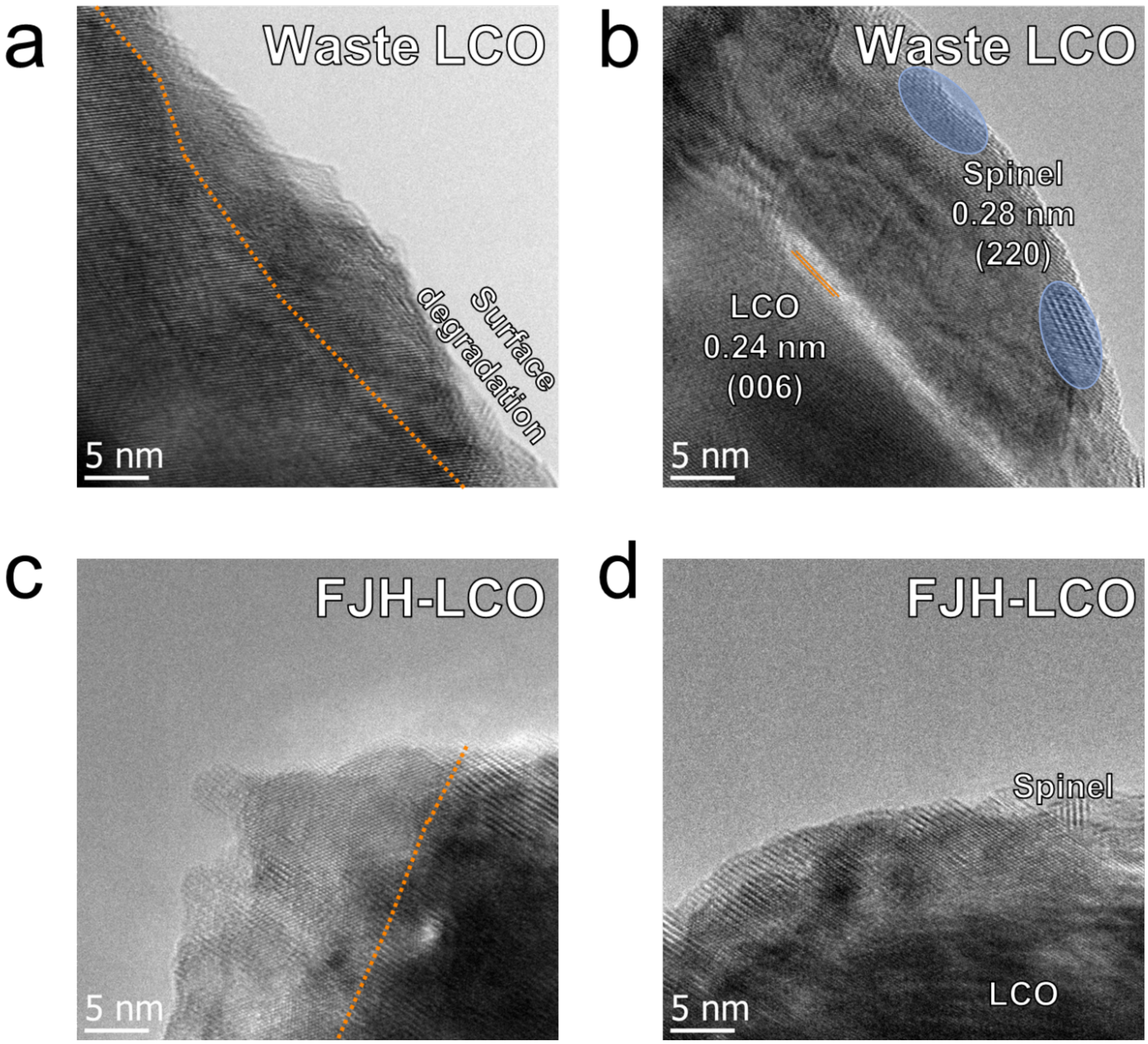

Supplementary Fig. 20. TEM results of waste LCO before and after FJH treatment. a,b, Waste LCO. **c,d,** FJH-LCO.

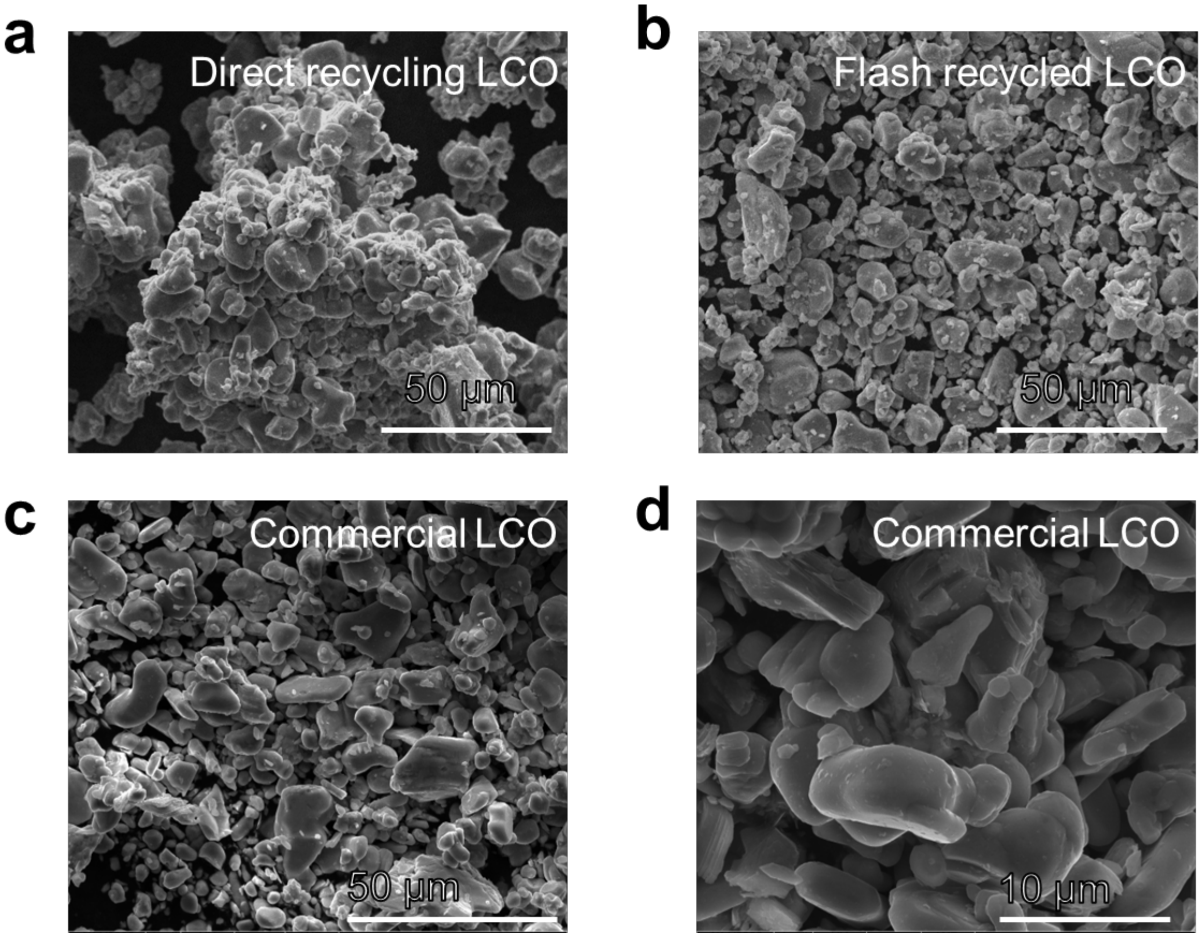

Supplementary Fig. 21. SEM images of different cathode materials. a, Direct recycling LCO. **b,** Flash recycled LCO. **c,d,** Commercial LCO.

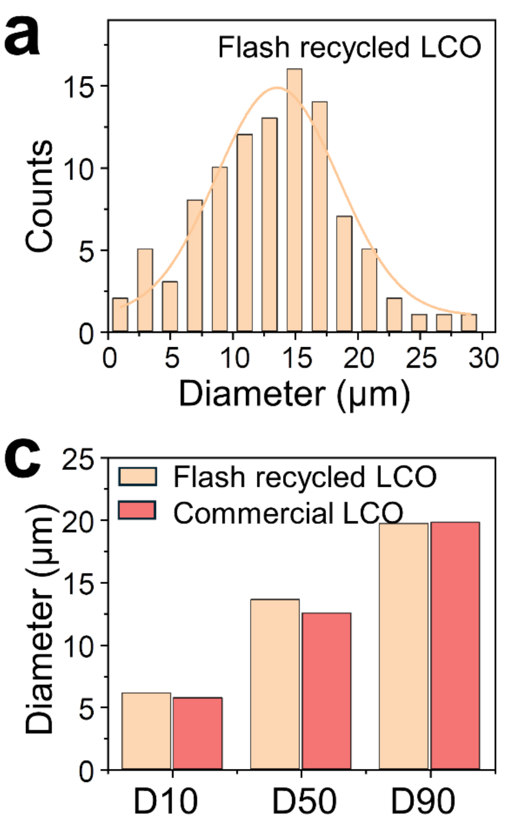

Supplementary Fig. 22. The particle sizes of different cathode materials. The size distribution of **a,** flash recycled LCO and **b,** **c**ommercial LCO. **c,** The size below which 10%, 50% or 90% of all particles are found for flash recycled LCO and **c**ommercial LCO (D10, D50 and D90).

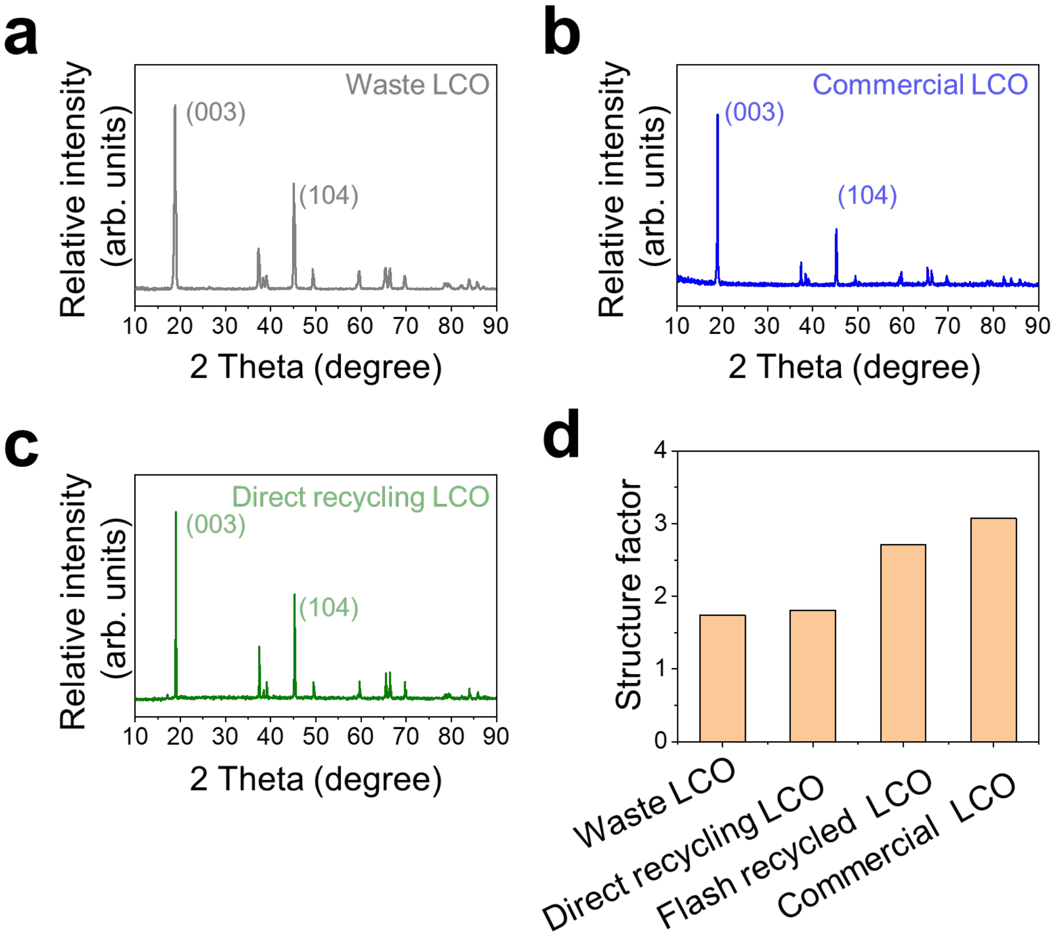

Supplementary Fig. 23. XRD results of various cathode materials. a, Waste LCO. **b,** Commercial LCO. **c,** Direct recycling LCO. **d,** Intensity ratio between (003) and (104) peaks (structure factor) for various cathode materials.

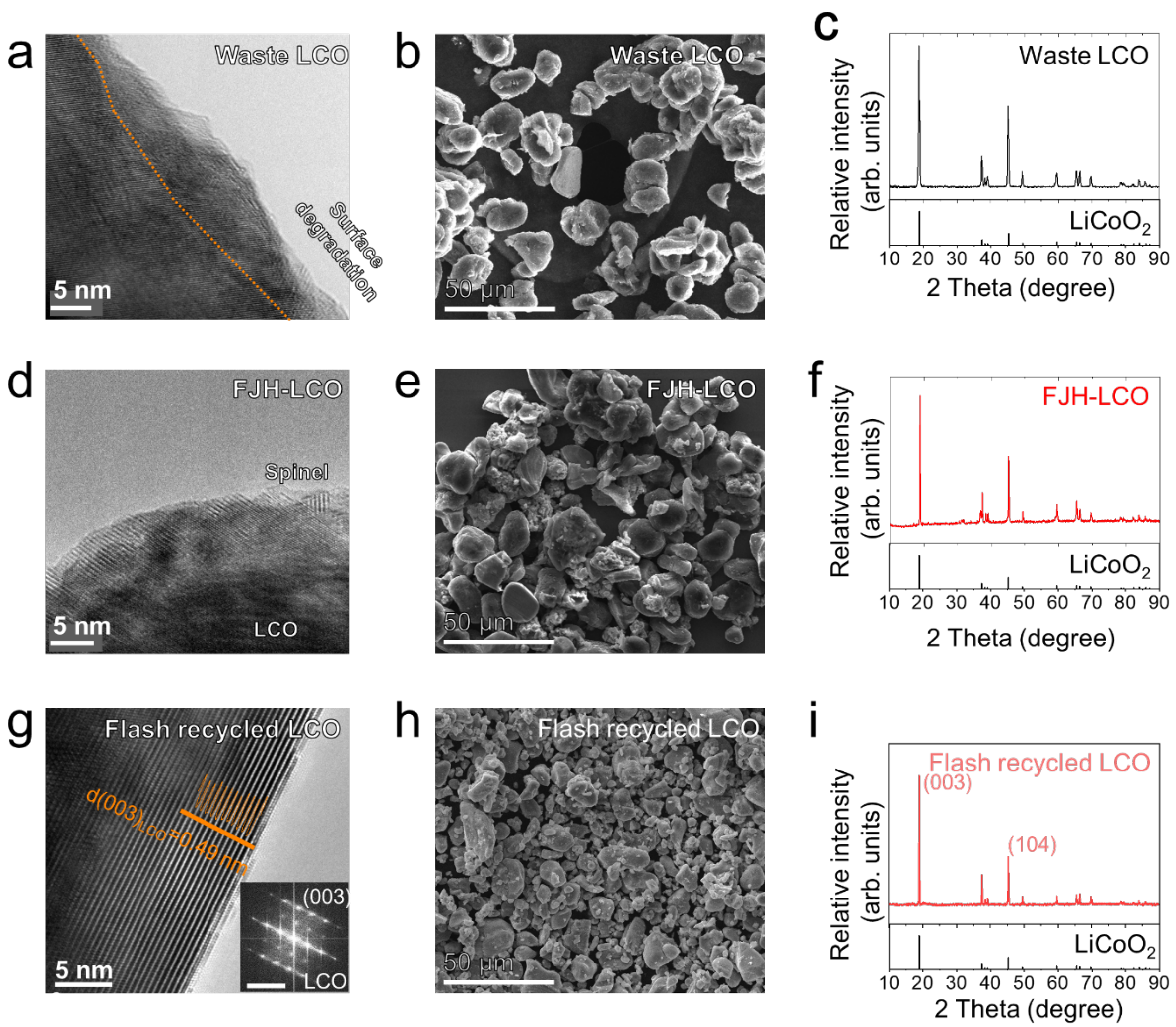

Supplementary Fig. 24. Structural characterization of cathode materials at different stages during flash recycling. a-c, Waste LCO. **d-f**, FJH-LCO. **g-i**, Flash recycled LCO. For **g**, the inset is the fast Fourier transform analysis of the indicated line.

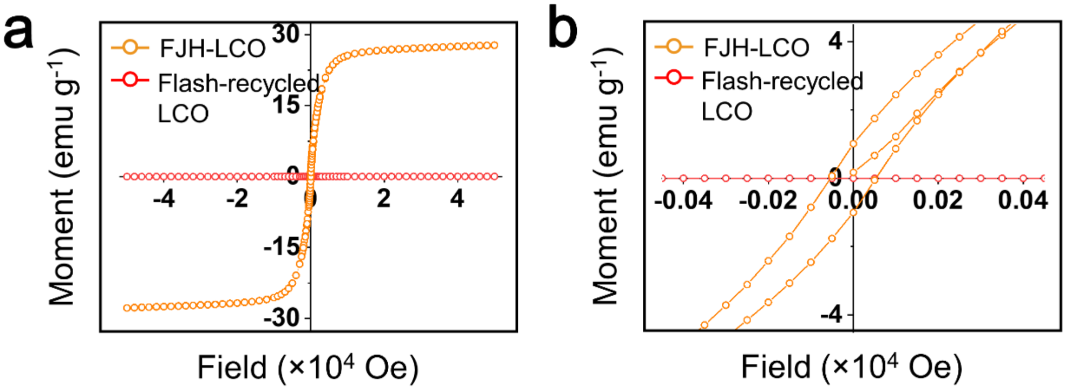

Supplementary Fig. 25. Magnetic response of various cathode materials. a, The room temperature (300 K) hysteresis loops for FJH-LCO (orange curve) and the flash recycled LCO (red curve). **b,** The behavior of the hysteresis loop around the origin for FJH-LCO (orange curve) and the flash recycled LCO (red curve).

Without the external magnetic field, the magnetic response was ~0.17 emu g^{-1} for FJH-LCO. The flash recycled LCO is not ferromagnetic without obvious magnetic response. The magnetic moment is 6.6×10^{-6} emu g^{-1} without external magnetic field. Since the total magnetic field for the Co_3O_4 is calculated to be ~70 emu g^{-1} as shown in Fig. 3j, the magnetic content of final product flash recycled LCO is calculated to be ~94 ppb.

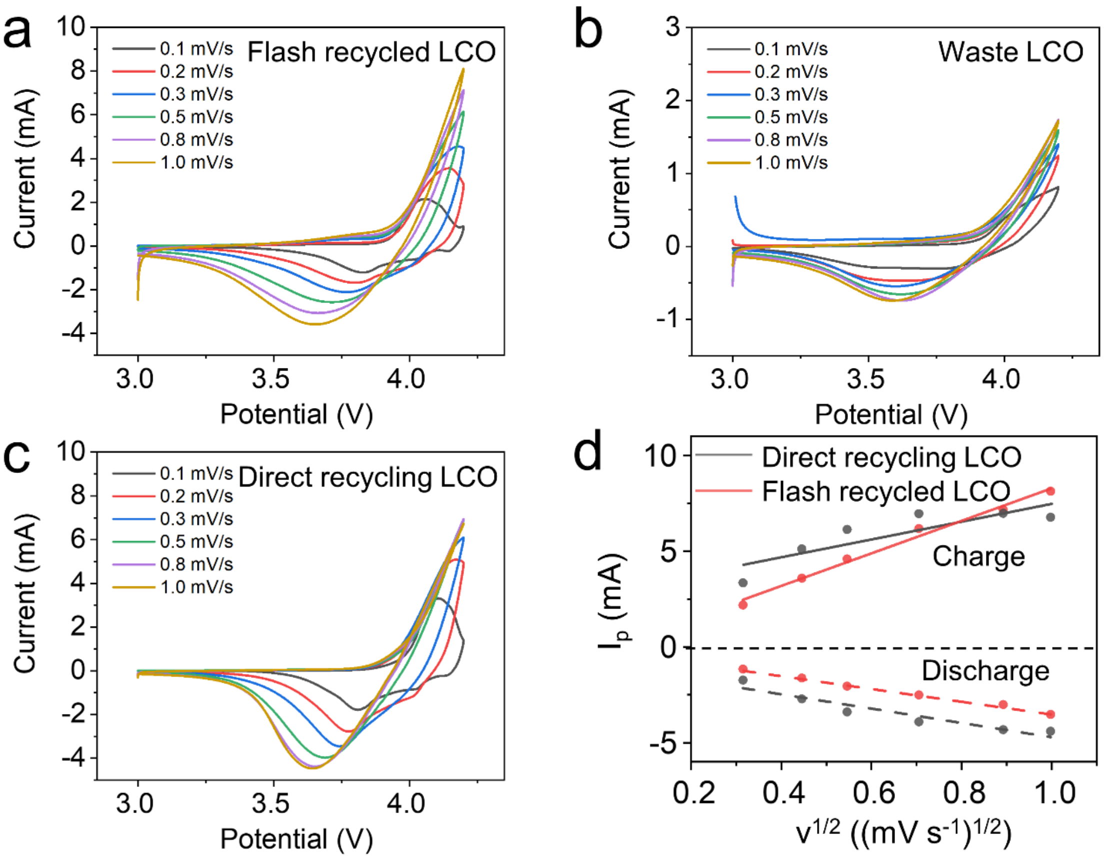

Supplementary Fig. 26. Cyclic voltammetry results of various cathode materials at different voltage scanning rates. a, Flash recycled LCO. **b,** Waste LCO. **c,** Direct recycling LCO. **d,** Comparison of diffusion coefficients of Li^+ in direct recycling LCO and flash recycled LCO. I_p: peak current.

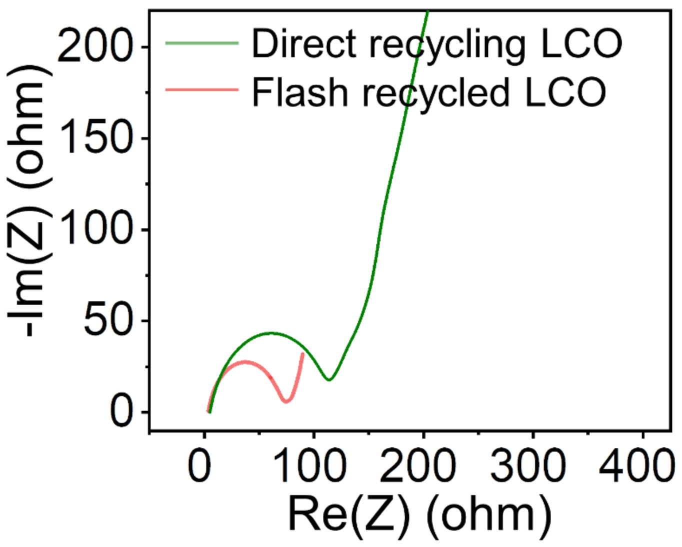

Supplementary Fig. 27. Electrochemical impedance spectroscopy of direct recycling LCO and flash recycled LCO.

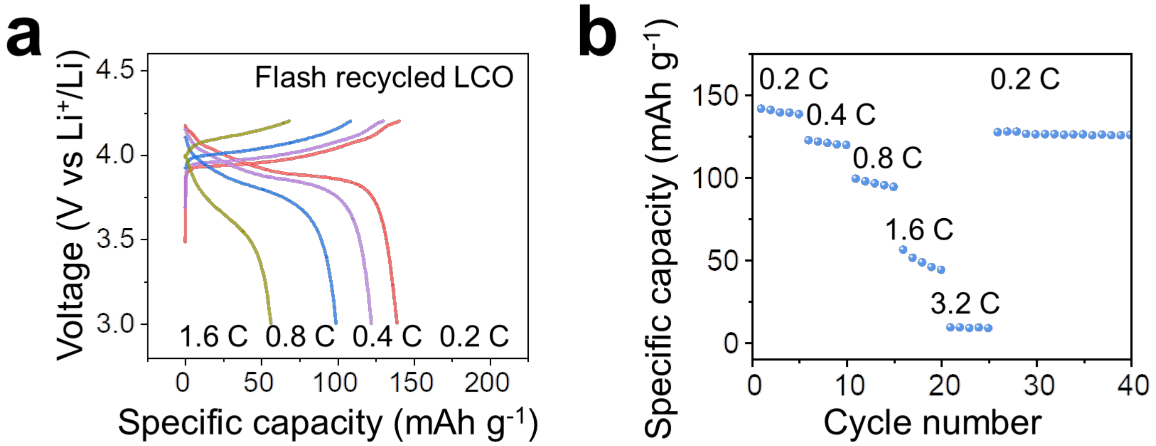

Supplementary Fig. 28. Rate performance of flash recycled LCO. a, Voltage profile and **b,** specific capacity of flash recycled LCO at different cycling rates.

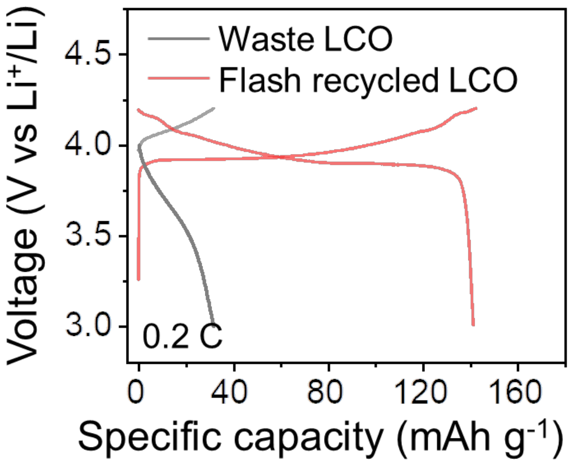

Supplementary Fig. 29. Electrochemical performance of flash recycled LCO. Voltage profile of flash recycled LCO and waste LCO at 0.2 C during the first cycle.

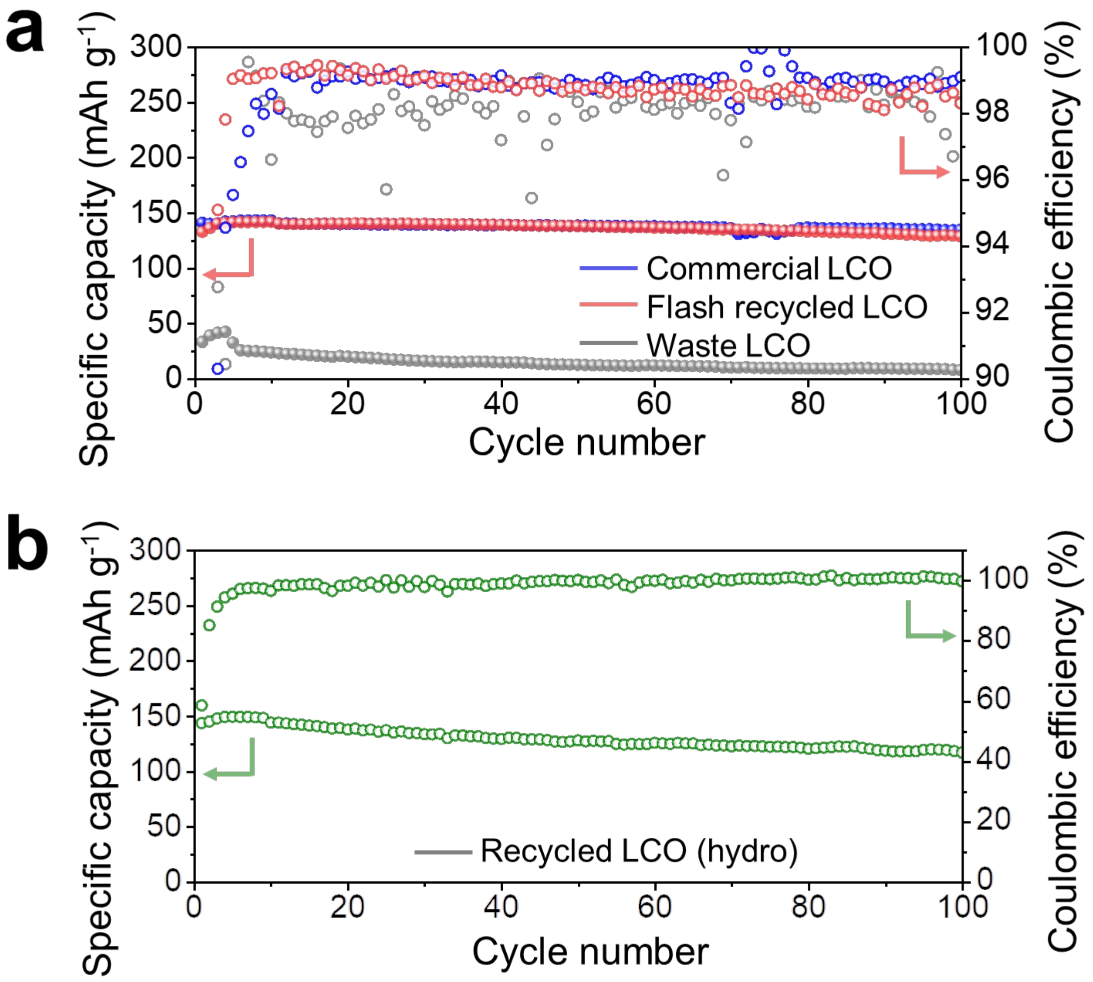

Supplementary Fig. 30. Electrochemical performance of flash recycled LCO. a, Cycling performance of commercial LCO, flash recycled LCO and waste LCO with a Li anode at 0.2 C. **b,** Cycling performance of recycled LCO from hydrometallurgical method with a Li anode at 0.2 C.

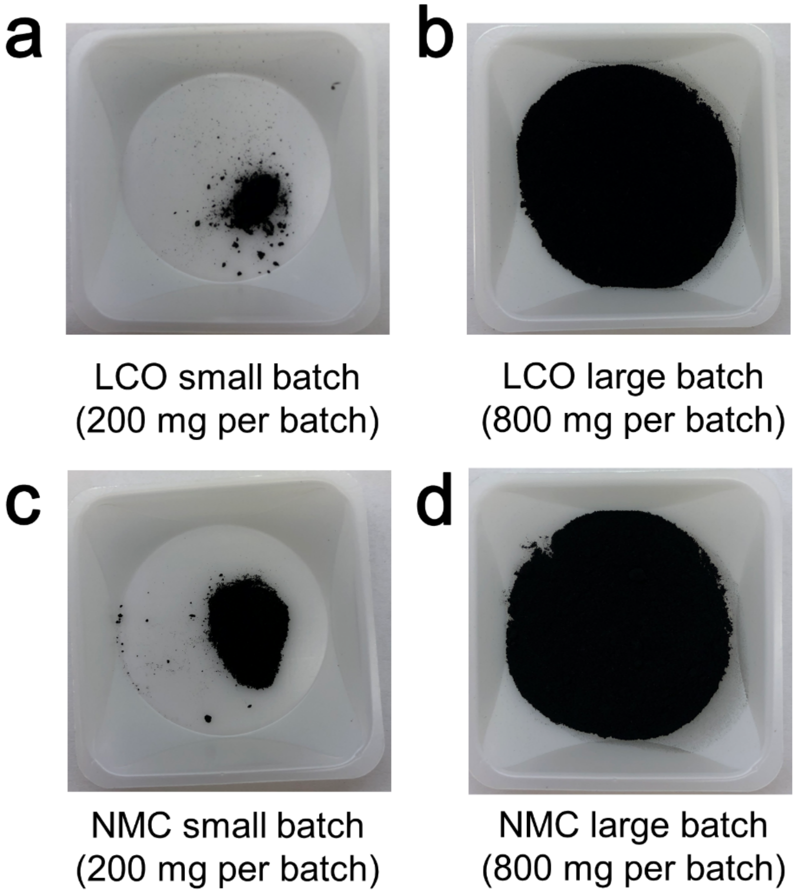

Supplementary Fig. 31. Optical images of flashed products in the gram-scale trials. a, LCO small batch. **b,** LCO large batch. **c,** NMC small batch. **d,** NMC big batch. For the small and large batches, the mass of reactants are 200 mg and 800 mg, respectively.

Supplementary Table 1. The composition of commercial lithium-ion batteries.

Materials	Price (USD per kg)	Estimated structure value (USD per kg)
Lithium	29.84	/
Cobalt	33.43	/
Nickel	20.89	/
Manganese	2.06	/
Iron	0.092	/
Phosphorus	0.34	/
Aluminum	2.16	/
Oxygen	0.20	/
$LiCoO_2$	47.77	25.46
NMC111	21.00	7.38
NMC532	20.00	7.01
NMC622	20.60	6.51
NMC811	22.00	7.58
$LiFePO_4$	14.00	12.04
NCA	21.50	5.96

Supplementary Table 2. Electrochemical performance of the regenerated cathode materials.

Methods	Cathode types	Areal capacity (mAh cm^{-2})	Specific capacity (mAh g^{-1})	Average capacity decay	Ref.
Flash recycling	LCO	~1.50	~143	~0.030% per cycle for 100 cycles	This work
Carbothermal shock	LCO	/	~133	~0.15% per cycle for 100 cycles	27
				~0.095% per cycle for 300 cycles	27
Direct recycling	LCO	~0.50	~139	~0.20% per cycle for 160 cycles	22
Direct recycling	LCO	~0.45	~148	~0.092% per cycle for 100 cycles	28
Direct recycling	Dual-doped LCO	~1.10	~220	~0.066% per cycle for 100 cycles	21
Direct recycling	LFP	0.34~0.51	~146	~0.032% per cycle for 400 cycles	20
Direct recycling	LFP	~3.23	~156	~0.006% per cycle for 100 cycles	16
Direct recycling	LMO	~1.48	~111	~0.13% per cycle for 100 cycles	23
Direct recycling	NMC	~1.73	~173	~0.16% per cycle for 100 cycles	24
Direct recycling	NMC	/	~146	~0.058% per cycle for 200 cycles	26
Hydrometallurgy	LCO	~0.80	~150	~0.31% per cycle for 40 cycles	9
Hydrometallurgy	NMC	/	~181	~0.070% per cycle for 100 cycles	30

Methods	Cathode types	Areal capacity (mAh cm^{-2})	Specific capacity (mAh g^{-1})	Average capacity decay	Ref.
Hydrometallurgy	NMC	~2.75	~130	~0.003% per cycle for 11600 cycles	29
Pyrometallurgy	LCO	~0.23	~145	~0.073% per cycle for 100 cycles	10
Pyrometallurgy	NMC	~3.20	~157	~0.051% per cycle for 100 cycles	13

Note:

"/" = Not mentioned in the literature reference.

Supplementary Table 3. The recovery efficiencies of battery metals by different recycle methods.

Method	Materials	Treatments	Recovery efficiency (%)		Ref
			Li	TM	
Pyrometallurgy	Spent LIBs (Li, Mn)	Vacuum pyrolysis (973 K, 0.5 h)	~82	-	36
Pyrometallurgy	Spent LIBs (Li, Co)	Vacuum pyrolysis (973 K, 0.5 h)	~83	-	36
Pyrometallurgy	Spent LIBs (Li, Co, Ni, Mn)	Vacuum pyrolysis (973 K, 0.5 h)	~66	-	36
Pyrometallurgy	Spent LIB (Li, Co, Ni, Mn)	High-temperature smelting (1473-1723 K, 2-6 h)	-	~90	4
Pyrometallurgy	Spent LIB (Li, Co)	Sulfation roasting (1073 K, 1 h)	~94	-	45
Pyrometallurgy	Spent LIB (Li, Co)	selective sulfating roasting (873 K, 1 h)	~1	~73	46
Pyrometallurgy	New cathode (Li, Co)	High-temperature smelting (1823 K, 12.5 h)	~100 (dust)[1]	~100	11
Pyrometallurgy	Spent LIBs (Li, Co, Ni, Mn)	Pretreatment (673 K, 2 h) De-coking (1023 K, 6 h) Smelting (1873 K, ~6.5 h)	~100 (dust)[1]	~100	11
Pyrometallurgy and hydrometallurgy	Spent LIBs (Li, Co, Ni, Mn)	Vacuum drying (353 K, 24 h) Calcination (873 K, 6 h) CH_3COOH leaching	~99	~100	13
Pyrometallurgy and hydrometallurgy	Spent LIBs (Li, Co)	Vacuum pyrolysis (873 K, 1.5 h) 2 M H_2SO_4 leaching	~99	~99	44
Hydrometallurgy	Spent LIBs (Li, Co)	4 M HCl leaching (353 K, 1 h)	~97	~97	43

Method	Materials	Treatments	Recovery efficiency (%)		Ref
			Li	TM	
Hydrometallurgy	Spent LIBs (Li, Co)	2 M H_2SO_4 + 5 Vol% H_2O_2 (348 K, 1 h)	~99	~70	42
Hydrometallurgy	Spent LIBs (Li, Co)	2 % H_3PO_4 + 2 Vol% H_2O_2 (363 K, 1 h)	~88	~99	41
Hydrometallurgy	Spent LIBs (Li, Co)	1 M HNO_3 + 1.7 Vol% H_2O_2 (348 K, 1 h)	~95	~95	40
Hydrometallurgy	Spent LIBs (Li, Co)	2 M citric acid + 0.6 g H_2O_2/g solid (343 K, 1.5 h)	~98	~96	39
Hydrometallurgy	Spent LIBs (Li, Co, Ni, Mn)	1.5 M lactic acid + 0.5 Vol% H_2O_2(343 K, 0.33 h)	~98	~98	38
Hydrometallurgy	Spent LIBs (Li, Co)	choline chloride and ethylene glycol (1:2) (453 K, 24 h)	~90	~50	37
Flash recycling	Spent LIBs (Li, Co)	Voltage 120 V (300 ms)	~94	~96	This work
Flash recycling	Spent LIBs (Li, Co, Ni, Mn)	Voltage 120 V (150 ms)	~95	~96	This work
Flash recycling	Mixed spent LIBs (Li, Co, Ni, Mn)	Voltage 150 V (300 ms)	~92	~96	This work

Note:

[1] = The Li source was from the flue dust which was needed to be collected from a cone-shape stainless steel cover placed at the outlet of the furnace.

- = ~0%

Supplementary Table 4. The composition of commercial lithium-ion batteries.

Materials	Ratio (wt%)	Materials	Ratio (wt%)
Cathode materials	~35.3	Graphite anode	~18.5
Conductive carbon	~2.4	Binder	~3.0
Aluminum	~8.1	Copper	~16.1
Electrolyte salts	~2.2	Electrolyte solvents	~12.0
Others	~2.4		

Supplementary Table 5. Life cycle inventory of various black mass recycling methods

Hydrometallurgical method[18]

Procedures	Input	Amount	Output	Amount	Notes	Ref
Discharging and collecting	**Spent batteries**	1.00 kg	**Spent batteries (100% SOD)**	1.00 kg	1 MJ electricity produces 0.13 kg GHG and 0.67 L water. 1 kg diesel produces 45.6 MJ energy. The data is estimated based on Everbatt 2020. ~5 wt% NaCl solution is used for the discharging process.	17
	Energy	0.03 MJ	GHG	0.004 kg		
	Water	0.52 L				
Shredding	**Spent batteries (100% SOD)**	1.00 kg	**Battery pieces**	0.88 kg	The battery pieces included the spent batteries without the organic solvents. The data is the average result from the industrial production (~10^4 tonne per year), the same below.	18
	Energy	0.38 MJ	GHG	0.050 kg		
	Water	0.26 L	Organic solvents	0.06 kg		
Low temperature calcination	**Battery pieces**	1.00 kg	**Calcined battery pieces**	0.94 kg	The purpose is to separate the active materials with current collectors, to decompose the binder, electrolyte residue, plastics, and SEI. The temperature is ~873 K for 2 h.	13
	Energy	0.20 MJ	GHG	0.526 kg		
	Water	0.14 L				
Wet granulation	**Calcined battery pieces**	1.00 kg	**Granulated battery pieces**	0.99 kg	The amount of water is estimated as ~20 wt% of the solid	70
	Energy	0.007 MJ	GHG	0.001 kg		
	Water	0.205 L				
Density separation	**Granulated battery pieces**	1.00 kg	**Spent active materials**	0.67 kg	The purpose is to separate the inactive materials, like steels, and the current collectors	18
	Energy	0.38 MJ	GHG	0.050 kg		
	Water	0.26 L	Current collector and case	0.31 kg		

Procedures	Input	Amount	Output	Amount	Notes	Ref
Froth flotation	**Spent active materials**	1.00 kg	**Spent cathode powder**	0.64 kg	The purpose is to separate the anode materials.	18
	Energy	0.67 MJ	GHG	0.087 kg		
	Water	0.45 L	Spent graphite	0.34 kg		
Acid leaching	**Spent cathode powder**	1.00 kg	**Leachate**	~50 kg	The average pulp density is ~2% and the concentration is ~4M HCl for the calculation in hydrometallurgical method. The density of 12M HCl is ~1.18 g cm^{-3} and 4M HCl is ~1.07 g cm^{-3}.	43
	Energy	0.11 MJ	GHG	0.014 kg		
	Water	30.99 L				
	12M HCl solution	18.02 kg				
Co-precipitation	**Leachate**	1.00 kg	**Transition metal hydroxides**	0.019 kg	The pH is adjusted to 10-11. The extra water is evaporated here to collect Li_2CO_3.	29
	Energy	0.11MJ	**Li_2CO_3**	0.007 kg		
	NaOH	0.15 kg	GHG	0.014 kg		
	Na_2CO_3	0.011 kg				
High-temperature resynthesize	**Transition metal hydroxides**	1.00 kg	**Resynthesized cathode**	1.05 kg	The molar ratio of n(Li):n(TM)=1.05 and the mixture is sintered at 723 K for 5 h and 1123 K for 14 h.	29
	**Li_2CO_3**	0.418 kg	GHG	2.698 kg		
	Energy	18.84 MJ				
	Water	12.63 L				

Pyrometallurgical method[18]

Procedures	Input	Amount	Output	Amount	Notes	Ref
Discharging and collecting	**Spent batteries**	1.00 kg	**Spent batteries (100% SOD)**	1.00 kg	1 MJ electricity produces 0.13 kg GHG and 0.67 L water. 1 kg diesel produces 45.6 MJ energy. The data is estimated based on Everbatt 2020. ~5 wt% NaCl solution is used for the discharging process.	17
	Energy	0.03 MJ	GHG	0.004 kg		
	Water	0.52 L				
Smelting	**Spent batteries (100% SOD)**	1.00 kg	**Matte (Co, Ni, Mn, Fe, Cu)**	0.39 kg	The purpose is to reduce the transition metals and remove all the impurities. The byproducts include the slag with Al, Ca, and Li, which requires the post-treatment to recycle the Li salts. The temperature is ~1873 K for 3 h.	18
	Energy	2.24 MJ	GHG	1.399 kg		
	Water	1.51 L	Slag (Al, Li, Ca)	0.406 kg		
	Slag formation reagent	0.30 kg				
Gas treatment	Energy	1.33 MJ	GHG	0.17 kg	For exhaust gas treatment	18
	Water	0.89 L				
Granulator	**Matte (Co, Ni, Mn, Fe, Cu)**	1.00 kg	**Fine metal particles**	0.99 kg		18
	Energy	0.007 MJ	GHG	0.001 kg		
	Water	0.005 L				
Acid leaching	**Fine metal particles**	1.00 kg	**Leachate**	20 kg	The average pulp density is ~5% and the concentration is ~1M HCl for the pyrometallurgical method. The density of 1M HCl is ~1.06 g cm^{-3}.	36
	Energy	0.11 MJ	GHG	0.014 kg		
	Water	17.31 L	Cu compounds	0.41 kg		
	12 M HCl solution	1.77 kg				

Procedures	Input	Amount	Output	Amount	Notes	Ref
Co-precipitation	**Leachate**	1.00 kg	**Transition metal hydroxides**	0.045 kg	The pH is adjusted to 10-11.	29
	Energy	0.11MJ	GHG	0.014 kg		
	NaOH	0.036 kg				
High-temperature resynthesize	**Transition metal hydroxides**	1.00 kg	**Resynthesized cathode**	1.05 kg	The molar ratio of n(Li):n(TM)=1.05 and the mixture is sintered at 723 K for 5 h and 1123 K for 14 h. The same as hydrometallurgical method.	29
	**Li_2CO_3**	0.418 kg	GHG	2.698 kg		
	Energy	18.84 MJ				
	Water	12.63 L				

Direct recycling method[18]

Procedures	Input	Amount	Output	Amount	Notes	Ref
Discharge and collecting	**Spent batteries**	1.00 kg	**Spent batteries (100% SOD)**	1.00 kg	1 MJ electricity produces 0.13 kg GHG and 0.67 L water. 1 kg diesel produces 45.6 MJ energy. The data is estimated based on Everbatt 2020. ~5 wt% NaCl solution is used for the discharging process.	17
	Energy	0.03 MJ	GHG	0.004 kg		
	Water	0.52 L				
Shredding	**Spent batteries (100% SOD)**	1.00 kg	**Battery pieces**	0.88 kg	The battery pieces included the spent batteries without the organic solvents. The data is the average result from the industrial production (~10^4 tonne per year), the same below.	18
	Energy	0.38 MJ	GHG	0.050 kg		
	Water	0.26 L	Organic solvents	0.06 kg		
Low temperature calcination	**Battery pieces**	1.00 kg	**Calcined battery pieces**	0.94 kg	The temperature is ~873 K for 2 h. The purpose is to decompose the binder, electrolyte residue, plastics, and SEI.	13
	Energy	0.20 MJ	GHG	0.526 kg		
	Water	0.14 L				
Screening	**Calcined battery pieces**	1.00 kg	**Spent active materials**	0.69 kg	The purpose is to separate the current collector and case from the spent active materials.	18
	Energy	0.11 MJ	Case and current collector	0.31 kg		
	Water	0.072 L	GHG	0.014 kg		
Froth flotation	**Spent active materials**	1.00 kg	**Spent cathode powder**	0.64 kg	The purpose is to separate the anode materials.	18
	Energy	0.67 MJ	GHG	0.087 kg		
	Water	0.45 L	Spent graphite	0.34 kg		

Procedures	Input	Amount	Output	Amount	Notes	Ref
Cathode re-lithiation	**Spent cathode powder**	1.00 kg	**Resynthesized cathode**	1.01 kg	The molar ratio of n(Li):n(TM)=1.10 and the mixture is sintered at 1073 K for 12 h.	18
	**Li_2CO_3**	0.12 kg	GHG	0.917 kg		
	Energy	7.05 MJ				
	Water	4.726 L				

Flash recycling method

Procedures	Input	Amount	Output	Amount	Notes	Ref
Discharge and collecting	**Spent batteries**	1.00 kg	**Spent batteries (100% SOD)**	1.00 kg	1 MJ electricity produces 0.13 kg GHG and 0.67 L water. 1 kg diesel produces 45.6 MJ energy. The data is estimated based on Everbatt 2020. ~5 wt% NaCl solution is used for the discharging process.	17
	Energy	0.03 MJ	GHG	0.004 kg		
	Water	0.52 L				
Disassembly and scrapping	**Spent batteries (100% SOD)**	1.00 kg	**Cathode waste**	0.37 kg	The spent active materials include spent cathode, anode powders and the electrolyte residue. Manual disassembly is considered to separate the spent active materials. The disassembly step can be achieved by a commercial core drill with a silicon carbide blade, which can reduce the manual disassembly cost.	9
	Energy	0.38 MJ	Anode waste	0.20 kg		
	Water	0.26 L	Case, separator, and current collector	0.28 kg		
			Organic solvent	0.06 kg		
			GHG	0.050 kg		
Flash Joule heating process and magnetic separation	**Cathode waste**	0.80 kg	**Flash-recycled cathode**	0.72 kg	The yield is ~90% for flash Joule heating reaction. The energy consumption is estimated based on the present experiment.	This work
	**Anode waste**	0.20 kg	Nonmagnetic product	0.18 kg		
	Energy	1.23 MJ	GHG	0.160 kg		
	Water	0.825 L				
Cathode re-lithiation	**Spent cathode powder**	1.00 kg	**Resynthesized cathode**	1.01 kg	The mixture is sintered at 1073 K for 12 h. The same as direct recycling method.	This work
	**Li_2CO_3**	0.20 kg	GHG	1.035 kg		
	Energy	7.05 MJ				
	Water	4.726 L				

Supplemental References

1. Rey, I., Vallejo, C., Santiago, G., Iturrondobeitia, M. & Lizundia, E. Environmental impacts of graphite recycling from spent lithium-ion batteries based on life cycle assessment. *ACS Sustainable Chem. Eng*. **9**, 14488−14501 (2021).
2. Xu, J. et al. A green and sustainable strategy toward lithium resources recycling from spent batteries. *Sci. Adv*. **8**, eabq7948 (2022).
3. Li, H. et al. A contact-electro-catalytic cathode recycling method for spent lithium-ion batteries. *Nat. Energy* **8**, 1137-1144 (2023).
4. Martínez, O. V., Valio, J., Aarnio, A. S., Reuter, M. & Guerrero, R. S. A critical review of lithium-ion battery recycling processes from a circular economy perspective. *Batteries* **5**, 68 (2019).
5. Jacoby, M. *Chem. Eng. News* **97** (2020). https://cen.acs.org/materials/energy-storage/time-serious-recycling-lithium/97/i28.
6. Natarajan, S. & Aravindan, V. Recycling strategies for spent Li-ion battery mixed cathodes. *ACS Energy Lett.* **3**, 2101-2103 (2018).
7. Salvatierra, R. V., Chen, W. & Tour, J. M. What can be expected from “anode-free” lithium metal batteries? *Adv. Energy Sustain. Res*. **2**, 2000110 (2021).
8. Ma, X. et al. Recycled cathode materials enabled superior performance for lithium-ion batteries. *Joule* **5**, 2955–2970 (2021).
9. Chen, W. et al. Battery metal recycling by flash Joule heating. *Sci. Adv*. **9**, eadh5131 (2023).
10. Tang, Y. et al. Recovery and regeneration of $LiCoO_2$-based spent lithium-ion batteries by a carbothermic reduction vacuum pyrolysis approach: Controlling the recovery of CoO or Co. *Waste Manag.* **97**, 140-148 (2019).

11. Hu, X., Mousa, E., Tian, Y. & Ye, G. Recovery of Co, Ni, Mn, and Li from Li-ion batteries by smelting reduction - Part I: A laboratory-scale study. *J. Power Sources* **483**, 228936 (2021).

12. Lv, W. et al. A critical review and analysis on the recycling of spent lithium-ion batteries. *ACS Sustain. Chem. Eng.* **6**, 1504–1521 (2018).

13. Zheng, Y. et al. Lithium nickel cobalt manganese oxide recovery *via* spray pyrolysis directly from the leachate of spent cathode scraps. *ACS Appl. Energy Mater.* **2**, 6952-6959 (2019).

14. Sethurajan, M. & Gaydardzhiev, S. Bioprocessing of spent lithium ion batteries for critical metals recovery – A review. *Resour. Conserv. Recycl.* **165**, 105225 (2021).

15. Jiao, M. et al. Recycling spent $LiNi_{1-x-y}Mn_xCo_yO_2$ cathodes to bifunctional NiMnCo catalysts for zinc-air batteries. *Proc. Natl. Acad. Sci.* **119**, e2202202119 (2022).

16. Xu, P. et al. Efficient direct recycling of lithium-ion battery cathodes by targeted healing. *Joule* **4**, 2609-2626 (2020).

17. Wang, M. et al. Summary of Expansions and Updates in GREET 2020 (2020 Report, Lemont, IL, USA).

18. Dai, Q. et al. Everbatt: A closed-loop battery recycling cost and environmental impacts model (2019); www.Anl.Gov/egs/everbatt.

19. Pender, J. P. et al. Electrode degradation in lithium-ion batteries. *ACS Nano* **14**, 1243–1295 (2020).

20. Ji, G. et al. Direct regeneration of degraded lithium-ion battery cathodes with a multifunctional organic lithium salt. *Nat. Commun.* **14**, 584 (2023).

21. Wang, J. et al. Sustainable upcycling of spent $LiCoO_2$ to an ultra-stable battery cathode at high voltage. *Nat. Sustain.* **6**, 797-805 (2023).

22. Sloop, S. et al. A direct recycling case study from a lithium-ion battery recall. *Sustain. Mater. Technol.* **25**, e00152 (2020).

23. Gao, H. et al. Efficient direct recycling of degraded $LiMn_2O_4$ cathodes by one-step hydrothermal relithiation. *ACS Appl. Mater. Interfaces* **12**, 51546-51554 (2020).

24. Wang, T. et al. Direct recycling of spent NCM cathodes through ionothermal lithiation. *Adv. Energy Mater.* **10**, 2001204 (2020).

25. Yang, Z., Zhang, J., Wu, Q., Zhi, L. & Zhang, W. Electrochemical regeneration of $LiFePO_4$/C cathode materials from spent lithium ion batteries. *J. Chin. Ceram. Soc.* **41**, 1051-1056 (2013).

26. Jiang, G. et al. Direct regeneration of $LiNi_{0.5}Co_{0.2}Mn_{0.3}O_2$ cathode from spent lithium-ion batteries by the molten salts method. *ACS Sustainable Chem. Eng.* **8**, 18138-18147 (2020).

27. Yin, Y. -C. et al. Rapid, direct regeneration of spent $LiCoO_2$ cathodes for Li-ion batteries. *ACS Energy Lett.* **8**, 3005-3012 (2023).

28. Shi, Y., Chen, G. & Chen, Z. Effective regeneration of $LiCoO_2$ from spent lithium-ion batteries: a direct approach towards high-performance active particles. *Green Chem.* **20**, 851-862 (2018).

29. Ma, X. et al. Recycled cathode materials enabled superior performance for lithium-ion batteries. *Joule* **5**, 2955-2970 (2021).

30. Wang, G. et al. Gradient-regeneration of $Li(Ni_{0.9}Co_{0.05}Mn_{0.05})O_2$ from spent $LiCoO_2$ lithium-ion battery. *J. Electrochem. Soc.* **167**, 160557 (2021).

31. Luong, D. X. et al. Gram-scale bottom-up flash graphene synthesis. *Nature* **577**, 647–651 (2020).

32. Chen, W. et al. Heteroatom-doped flash graphene. *ACS Nano* **16**, 6646–6656 (2022).

33. Algozeeb, W. A. et al. Flash graphene from plastic waste. *ACS Nano* **14**, 15595–15604 (2020).

34. Chen, W. et al. Flash recycling of graphite anodes. *Adv. Mater*. **35**, 2207303 (2023).

35. Deng, B. et al. Phase controlled synthesis of transition metal carbide nanocrystals by ultrafast flash Joule heating. *Nat. Commun*. **13**, 262 (2022).

36. Xiao, J., Li, J. & Xu, Z. Novel approach for in situ recovery of lithium carbonate from spent lithium ion batteries using vacuum metallurgy. *Environ. Sci. Technol*. **51**, 11960-11966 (2017).

37. Tran, M. K., Rodrigues, M.-T. F., Kato, K., Babu, G. & Ajayan, P. M. Deep eutectic solvents for cathode recycling of Li-ion batteries. *Nat. Energy* **4**, 339-345 (2019).

38. Li, L. et al. Sustainable recovery of cathode materials from spent lithium-ion batteries using lactic acid leaching system. *ACS Sustain. Chem. Eng*. **5**, 5224–5233 (2017).

39. Chen, X., Luo, C., Zhang, J., Kong, J. & Zhou, T. Sustainable recovery of metals from spent lithium-ion batteries: A green process. *ACS Sustain. Chem. Eng*. **3**, 3104-3113 (2015).

40. Lee, C. K. & Rhee, K. -I. Preparation of $LiCoO_2$ from spent lithium-ion batteries. *J. Power Sources* **109**, 17-21 (2002).

41. Pinna, E. G., Ruiz, M. C., Ojeda, M. W. & Rodriguez, M. H. Cathodes of spent Li-ion batteries: Dissolution with phosphoric acid and recovery of lithium and cobalt from leach liquors. *Hydrometallurgy* **167**, 66-71 (2017).

42. Jha, M. K. et al. Recovery of lithium and cobalt from waste lithium ion batteries of mobile phone. *Waste Manag. 33*, 1890-1897 (2013).

43. Zhang, P., Yokoyama, T., Itabashi, O., Suzuki, T. M. & Inoue, K. Hydrometallurgical process for recovery of metal values from spent lithium-ion secondary batteries. *Hydrometallurgy* **47**, 259–271 (1998).

44. Sun, L. & Qiu, K. Vacuum pyrolysis and hydrometallurgical process for the recovery of valuable metals from spent lithium-ion batteries. *J. Hazard. Mater.* **194**, 378-384 (2011).

45. Li, N. et al. Aqueous leaching of lithium from simulated pyrometallurgical slag by sodium sulfate roasting. *RSC Adv*. **9**, 23908-23915 (2019).

46. Wang, D., Zhang, X., Chen, H. & Sun, J. Separation of Li and Co from the active mass of spent Li-ion batteries by selective sulfating roasting with sodium bisulfate and water leaching. *Miner. Eng.* **126**, 28-35 (2018).

47. Zhang, R. et al. Systematic study of Al impurity for NCM622 cathode materials. *ACS Sustainable Chem. Eng*. **8**, 9875-9884 (2020).

48. Peng, C. et al. Role of impurity copper in Li-ion battery recycling to $LiCoO_2$ cathode materials. *J. Power Sources* **450**, 227630 (2020).

49. Vila, R. A., Huang, W. & Cui, Y. Nickel impurities in the solid-electrolyte interphase of lithium-metal anodes revealed by cryogenic electron microscopy. *Cell Rep. Phys. Sci.* **1**, 100188 (2020).

50. Li, T. et al. Direct and rapid high-temperature upcycling of degraded graphite. *Adv. Funct. Mater.* **33**, 2302951 (2023).

51. Deng, B. et al. Rare earth elements from waste. *Sci. Adv*. **8**, eabm3132 (2022).

52. Dong, Q. et al. Programmable heating and quenching for efficient thermochemical synthesis. *Nature* **605**, 470–476 (2022).

53. Dai, T. et al. Synergy of lithium, cobalt, and oxygen vacancies in lithium cobalt oxide for airborne benzene oxidation: A concept of reusing electronic wastes for air pollutant removal. *ACS Sustain. Chem. Eng*. **7**, 5072-5081 (2019).

54. Apátiga, L. M. & Castaño, V. M. Magnetic behavior of cobalt oxide films prepared by pulsed liquid injection chemical vapor deposition from a metal-organic precursor. *Thin Solid Films* **496**, 576-579 (2006).

55. Moro, F., Tang, S. V. Y., Tuna, F. & Lester, E. Magnetic properties of cobalt oxide nanoparticles synthesised by a continuous hydrothermal method. *J Magn. Magn* **348**, 1-7 (2013).

56. Zhang, D. et al. Controllable fabrication and magnetic properties of double-shell cobalt oxides hollow particles. *Sci. Rep*. **5**, 8737 (2015).

57. Huang, K. -P. et al. Magnetic impurity effects on self-discharge capacity, cycle performance, and rate capability of $LiFePO_4$/C composites. *J. Solid State Electrochem*. **21**, 1767–1775 (2017).

58. Wyss, K. M. et al. Upcycling end-of-life vehicle waste plastic into flash graphene. *Comms. Eng.* **1**, 3 (2022).

59. Liu, Y. et al. 3D cube-maze-like Li-rich layered cathodes assembled from 2D porous nanosheets for enhanced cycle stability and rate capability of lithium-ion batteries. *Adv. Energy Mater.* **10**, 1903139 (2020).

60. Cheng, X. et al. Regulating surface and grain-boundary structures of Ni-rich layered cathodes for ultrahigh cycle stability. *Small* **16**, 1906433 (2020).

61. Yu, J. et al. Mechanochemical upcycling of spent $LiCoO_2$ to new $LiNi_{0.80}Co_{0.15}Al_{0.05}O_2$ battery: An atom economy strategy. *Proc. Natl. Acad. Sci. U.S.A*. **120**, e2217698120 (2023).

62. Zhu, G. et al. High energy density hybrid lithium-ion capacitor enabled by Co_3ZnC@ N-doped carbon nanopolyhedra anode and microporous carbon cathode. *Energy Storage Mater*. **14**, 246-252 (2018).

63. Liu, K., Liu, L., Tan, Q. & Li, J. Selective extraction of lithium from a spent lithium iron phosphate battery by mechanochemical solid-phase oxidation. *Green Chem*. **23**, 1344-1352 (2021).

64. Ma, L. et al. Cerium oxide nanocrystal embedded bimodal micromesoporous nitrogen-rich carbon nanospheres as effective sulfur host for lithium–sulfur batteries. *ACS Nano* **11**, 7274-7283 (2017).

65. Song, D. et al. Advancing recycling of spent lithium-ion batteries: From green chemistry to circular economy. *Energy Storage Mater*. **61**, 102870 (2023).

66. Eddy, L. et al. Laboratory kilogram-scale graphene production from coal. DOI: 10.26434/chemrxiv-2023-7z1s2 (2023).

67 Dong, S. et al. Ultra-fast, low-cost, and green regeneration of graphite anode using flash joule heating method. *EcoMat*. **4**, e12212 (2022).

68 Luo, J. et al. Recycle spent graphite to defect-engineered, high-power graphite anode. *Nano Res*. **16**, 4240-4245 (2023).

69. Scaleup of FJH for graphene synthesis; www.universalmatter.com/ [accessed 18 September 2023].

70. Miwa, A., Yajima, T. & Itai, S. Prediction of suitable amount of water addition for wet granulation. *Int. J. Pharm*. **195**, 81–92 (2000).
