# Supplementary material for: Nondestructive flash cathode recycling
Source: Nat Commun. 2024 Jul 24;15:6250. doi: 10.1038/s41467-024-50324-x (PMC11269590; doi:10.1038/s41467-024-50324-x)
Supplement: Supplementary file 3 — Description of Additional Supplementary Files [file 41467_2024_50324_MOESM3_ESM.pdf]

File name: Supplementary Video 1.mp4

Tribology Experiment Video Description: In this video, we demonstrate the tribological experimental procedure to obtain the coefficient of friction.
